# Discovery of a novel ALK/ROS1/FAK inhibitor, APG-2449, in preclinical non-small cell lung cancer and ovarian cancer models

**DOI:** 10.1186/s12885-022-09799-4

**Published:** 2022-07-11

**Authors:** Douglas D. Fang, Ran Tao, Guangfeng Wang, Yuanbao Li, Kaixiang Zhang, Chunhua Xu, Guoqin Zhai, Qixin Wang, Jingwen Wang, Chunyang Tang, Ping Min, Dengkun Xiong, Jianyong Chen, Shaomeng Wang, Dajun Yang, Yifan Zhai

**Affiliations:** 1 Ascentage Pharma (Suzhou) Co., Ltd, 68 Xinqing Road, Suzhou, 215214 China; 2 Pharmacology and Medicinal Chemistry, Michigan Center for Therapeutic Innovation, University of Michigan, 1600 Huron Parkway NCRC/Building 520 Room 1245, Ann Arbor, MI 48109 USA; 3 Department of Experimental Research, State Key Laboratory of Oncology in South China, Collaborative Innovation Center for Cancer Medicine, Sun Yat-sen University Cancer Center, Guangzhou, 510275 China

**Keywords:** Anaplastic lymphoma kinase (ALK), Focal adhesion kinase (FAK), ROS proto-oncogene 1 receptor tyrosine kinase (ROS1), Solid tumors, Targeted therapies

## Abstract

**Background:**

Tyrosine kinase inhibitors (TKIs) are mainstays of cancer treatment. However, their clinical benefits are often constrained by acquired resistance. To overcome such outcomes, we have rationally engineered APG-2449 as a novel multikinase inhibitor that is highly potent against oncogenic alterations of anaplastic lymphoma kinase (*ALK*), ROS proto-oncogene 1 receptor tyrosine kinase (*ROS1*), and focal adhesion kinase (*FAK*). Here we present the preclinical evaluation of APG-2449, which exhibits antiproliferative activity in cells carrying *ALK* fusion or secondary mutations.

**Methods:**

KINOMEscan® and LANCE TR-FRET were used to characterize targets and selectivity of APG-2449. Water-soluble tetrazolium salt (WST-8) viability assay and xenograft tumorigenicity were employed to evaluate therapeutic efficacy of monotherapy or drug combination in preclinical models of solid tumors. Western blot, pharmacokinetic, and flow cytometry analyses, as well as RNA sequencing were used to explore pharmacokinetic–pharmacodynamic correlations and the mechanism of actions driving drug combination synergy.

**Results:**

In mice bearing wild-type or *ALK/ROS1*-mutant non-small-cell lung cancer (NSCLC), APG-2449 demonstrates potent antitumor activity, with correlations between pharmacokinetics and pharmacodynamics in vivo. Through FAK inhibition, APG-2449 sensitizes ovarian xenograft tumors to paclitaxel by reducing CD44^+^ and aldehyde dehydrogenase 1-positive (ALDH1^+^) cancer stem cell populations, including ovarian tumors insensitive to carboplatin. In epidermal growth factor receptor (*EGFR)*-mutated NSCLC xenograft models, APG-2449 enhances EGFR TKI-induced tumor growth inhibition, while the ternary combination of APG-2449 with EGFR (osimertinib) and mitogen-activated extracellular signal-regulated kinase (MEK; trametinib) inhibitors overcomes osimertinib resistance. Mechanistically, phosphorylation of ALK, ROS1, and FAK, as well as their downstream components, is effectively inhibited by APG-2449.

**Conclusions:**

Taken together, our studies demonstrate that APG-2449 exerts potent and durable antitumor activity in human NSCLC and ovarian tumor models when administered alone or in combination with other therapies. A phase 1 clinical trial has been initiated to evaluate the safety and preliminary efficacy of APG-2449 in patients with advanced solid tumors, including *ALK*^+^ NSCLC refractory to earlier-generation ALK inhibitors.

**Trial registration:**

Clinicaltrial.gov registration: NCT03917043 (date of first registration, 16/04/2019) and Chinese clinical trial registration: CTR20190468 (date of first registration, 09/04/2019).

**Supplementary Information:**

The online version contains supplementary material available at 10.1186/s12885-022-09799-4.

## Significance

In preclinical experiments, APG-2449 is active against treatment-resistant (e.g., lung, ovarian) tumors. If confirmed in clinical trials, these findings may support the role of APG-2449 as a novel therapy for these conditions.

## Background

Molecular targeted therapies (e.g., tyrosine kinase inhibitors [TKIs]), have achieved clinical successes when directed against strong oncogenic drivers, such as anaplastic lymphoma kinase (*ALK*) rearrangement and epidermal growth factor receptor (*EGFR*) mutation. Despite initial responses, patients almost inevitably develop TKI resistance by acquiring or expanding resistance mutations [1], within 12–24 months for crizotinib [2]. Treatment with crizotinib, an ALK inhibitor and one of the earliest validated TKIs, results in an overall response rate of ~ 60% in patients with *ALK*-positive (*ALK*^*+*^) non-small-cell lung cancer (NSCLC) [2, 3]. Extensive efforts have been undertaken to develop novel TKIs that deepen treatment responses and overcome drug resistance, culminating in discovery of second-generation (2G) TKIs ceritinib, alectinib, and brigatinib, as well as third-generation (3G) agent lorlatinib.

Our efforts to develop a novel TKI resulted in APG-2449, which demonstrated activity against ALK, ROS proto-oncogene 1 receptor tyrosine kinase (ROS1), and focal adhesion kinase (FAK) [4]. *ROS1* gene fusions account for ~ 1 to 2% of all cases of NSCLC [5]. FAK is a ubiquitous intracellular nonreceptor tyrosine kinase localized at focal adhesions and is a key regulator of cellular adhesion, migration, and proliferation [6, 7]. *FAK* gene overexpression or amplification occurs in several malignancies [8] and is linked to tumor progression, metastasis, drug resistance, and a poor prognosis [9–11]. To date, several FAK inhibitors such as defactinib (VS-6063) are under clinical development. Despite preclinical antitumor effects [12–16], objective clinical responses have not been achieved [17–19], apart from extending progression-free survival and stabilizing disease. Pharmaceutical development strategies have largely shifted to combinations of TKIs with chemotherapeutics, targeted therapies, and/or immune modulators for drug-resistant cancers, especially chemotherapy-resistant ovarian cancer [10] and EGFR TKI-resistant NSCLC [20, 21]. To advance these initiatives, we conducted in vitro and in vivo studies on the effects of multikinase inhibitor APG-2449, alone or with available therapies, in overcoming TKI resistance observed in *ALK-*, *ROS1-*, and *FAK-*mutated tumors.

## Materials and methods

### Mice

Cancer cell-line-derived xenograft (CDX) studies were conducted in the animal facility of Shanghai Institute of Planned Parenthood Research (Shanghai) or GenePharma Co., Ltd. (Suzhou) [22, 23]. Patient-derived xenograft (PDX) studies were conducted at: (1) WuXi AppTec (Suzhou; LU-01-0582R), (2) Shanghai LIDE (Lab for Innovated Diagnosis and Experimental Therapeutics) Biotech Co., Ltd. (Shanghai; LD1–0006-215,676 and LD1–0006-390,637) and (3) CrownBio (Taicang, China; OV0243, OV1385, OV1396, OV1658, OV2018, and OV2423). Protocols involving care and use of animals were approved by Institutional Animal Care and Use Committees.

### Kinase activity profile

Kinase activity inhibition by APG-2449 100 nM was profiled against 468 human kinases via LeadHunter® Drug Discovery Services Panels (Eurofins DiscoverX Products, LLC, Fremont, CA). Kinase specificity was analyzed and visualized using TREEspot™ software (DiscoverX).

### Lanthanide chelate excite time-resolved fluorescence resonance energy transfer (LANCE TR-FRET) assay

The cytoplasmic domain (amino acid residues 1058–1620) of human wild-type (*wt*)-ALK protein expressed as an N-terminal glutathione S-transferase fusion protein was purchased from Carna Biosciences (Kobe, Japan). Mutated proteins from mutant *ALK* were expressed in SF9 insect cells with N-terminal tags cleaved after purification. Kinase activities of all enzymes were assessed using a LANCE TR-FRET assay kit (PerkinElmer, Waltham, MA), as previously reported [24].

### Cell lines and reagents

Purchased human cancer cell lines included: (1) ES-2, HO-8910 PM and SW620 (Shanghai Institute of Biochemistry and Cell Biology); (2) HCC827, NCI-H1650, NCI-H1975, HepG2, and IMR-32 (American Type Culture Collection; Manassas, VA); and (3) OVCAR-3 (China Center for Type Culture Collection; Wuhan). All other human lines were obtained from Cobio Biosciences (Nanjing, China). Murine pro-B cell lines BaF3 and BaF3_*EML4-ALK*^*G1202R*^ were purchased from KYinoo (Beijing). Other BaF3-engineered cell lines were constructed internally. An osimertinib-resistant parental cell (PC-9/OR) line was established by exposing PC-9 cells to escalating concentrations of osimertinib for 3 months.

APG-2449 was synthesized by Ascentage Pharma. Alectinib, ceritinib, crizotinib, defactinib, erlotinib, lorlatinib, osimertinib, and trametinib, as well as paclitaxel and carboplatin, were purchased from Selleckchem (Houston, TX). Ensartinib was purchased from Birdo Tech (Shanghai).

### Cell proliferation assay

Cell viability was determined using the water-soluble tetrazolium salt (WST-8) assay (Cell counting Kit-8, Shanghai Life iLab). Cell viability was calculated as follows, where “RLU” signifies relative light units:

$$Cell\ viability=\frac{\left( mean\ RLU\ sample- mean\ RLU\ blank\right)}{\left( RLU\ cell\ control- RLU\ blank\right)}\times 100$$

Half-maximal inhibitory concentration (IC_50_) values were calculated using Prism (GraphPad, San Diego, CA).

### ALDEFLUOR assay and flow cytometry

SKOV-3 ovarian cancer cells were treated for 72 hours, and ALDH1 was analyzed using an ALDEFLUOR™ Kit (STEMCELL Technologies Canada Inc., Vancouver, BC; Cat. #01700) according to manufacturer instructions. Incubation with ALDH inhibitor 4-diethylaminobenzaldehyde (DEAB) was included as a negative control. ALDH1-positive (ALDH1^+^) cells were determined in relation to controls exposed to DEAB. To assess CD44 expression, we stained cells with CD44-phycoerythrin (PE)-conjugated antibody (Invitrogen™, Carlsbad, CA). Fluorescent intensities of ALDH1^+^ and CD44^+^ cells were acquired on an Attune NxT flow cytometer (Life Technology, Carlsbad, CA) and analyzed using FlowJo software (BD Biosciences, San Jose, CA).

### In vivo antitumor activity in mouse xenograft models

To establish CDX models, we subcutaneously implanted immunodeficient mice (Vital River Laboratory Animal Technology Co., Ltd., Beijing) with 0.5 to 5 × 10^7^ cancer cells mixed with 30% Corning® Matrigel® matrix per animal. PDX model LD1–0006-390,637 was derived from a patient who experienced relapsed NSCLC and harbored concurrent *ALK*^*L1196M_G1202R*^ mutations. Crizotinib-resistant NSCLC PDX model LU-01-0582R was established by administering crizotinib to tumor-bearing mice through 6 implantations and drug exposure cycles. No secondary *ALK* mutations were identified in LU-01-0582R xenografts.

A range of compounds were administered orally (PO) once daily (QD). APG-2449 was dissolved in an 80% 10 mM monobasic sodium phosphate vehicle (Titan Scientific Co., Ltd., Shanghai) plus 20% propylene glycol (Sigma-Aldrich). Alectinib, ceritinib, crizotinib, ensartinib, and lorlatinib were dissolved in 0.5% (hydroxypropyl)methyl cellulose (Sigma-Aldrich Cat. # 09963) plus 0.2% TWEEN 80 (Sigma-Aldrich). Osimertinib was dissolved in 20% polyethylene glycol 400 (PEG-400; Sigma-Aldrich) and erlotinib in 2% TWEEN 80 (Sigma-Aldrich). Trametinib was dissolved in a solvent composed of 1% carboxymethylcellulose, 0.5% TWEEN 80, and 0.5% methylcellulose. Defactinib was dissolved in 10% PEG 400 plus 5% ethanol (Sigma-Aldrich) in 1× phosphate-buffered saline (Genom Biotech; Hangzhou, China) and administered PO twice daily. Paclitaxel dissolved in saline was administered intraperitoneally once weekly.

The following were computed as previously reported (23): mean ± SEM tumor volumes, percent changes in body weight, tumor growth inhibition in treatment/control (T/C; %), modified Response Evaluation Criteria in Solid Tumors (mRECIST) [25] objective response rate (ORR %), and disease control rate (DCR, %).

### Pharmacokinetic (PK) analysis

Systemic and tumor tissue APG-2449 exposures were measured by liquid chromatography tandem mass spectrometry (LC/MS/MS) using an Exion high-performance LC system (AB Sciex, Ontario, Canada) coupled to an Atmospheric Pressure Ionization (API 5500) MS (AB Sciex) equipped with an API electrospray ionization source.

### Western blot analysis

Western blot analysis was performed as described previously [23]. Blots were cut prior to hybridization with the antibodies. Images of original cut blots with membrane edge visible are included (where applicable) in the Supplementary Material. Protein expression was quantitated using ImageJ (Image Processing and Analysis in Java) software (GitHub; Wayne Rasband, US National Institute of Mental Health) and normalized with β-actin to compute relative fold changes of proteins (vs. vehicle control). Antibody information is enumerated in Supplementary Table S1.

### Immunohistochemistry (IHC)

PDX tumor tissues were cut to a thickness of 4 μm and stained with antibodies against FAK (Merck Millipore/Merck KgaA, Burlington, MA; Cat. #05–537; 1:800 dilution), P-FAK (Cat. #SAB4504148; 1:200), CD44 (Abcam, Shanghai; Cat. #Ab51037; 1:400), or E-cadherin (Cell Signaling Technology; Cat. #3195 s; 1:400).

IHC staining was performed on the BOND RX automated IHC & In Situ Hybridization system with the Detection System Horseradish Peroxidase (HRP) Polymer Kit (each from Leica Biosystems, Danvers, MA). Stained sections were scanned using a Hamamatsu Photonics NanoZoomer 2.0-HT Image system (Hamamatsu, Japan) at 40× magnification. Images were analyzed on a HALO® image analysis platform (v3.0.311.363; Indica Labs, Albuquerque, NM). IHC staining intensity was scored as 0 (negative), 1^+^ (weak), 2^+^ (medium), or 3^+^ (strong). The percentages of tumor cells at different intensity levels were evaluated as:

$$\mathrm{H}-\mathrm{Score}=\left(\%\mathrm{at}\ 0\right)\times 0+\left(\%\mathrm{at}\ 1\right)\times 1+\left(\%\mathrm{at}\ 2\right)\times 2+\left(\%\mathrm{at}\ 3\right)\times 3.$$

### RNA-sequencing (RNA-seq)

RNA-seq of ovarian PDX tumors was performed using an Illumina NovaSeq 6000 system (Crown Bioscience Inc., San Diego, CA). Differential expression analysis was carried out using the Empirical Analysis of Digital Gene Expression Data in R (edgeR) package (Bioconductor Open-Source Software for Bioinformatics; Stanford, CA). Significantly differentially expressed genes were defined as a false-discovery rate of < 0.05 and a fold change of ≥ |2|.

### Statistical analysis

To assess statistical significance of differences between treatment groups, we conducted two-tailed Student *t*-tests and one-way analyses of variance followed by Games-Howell’s post-test (for multiple comparisons). The sample size calculation was based on a resource equation [26]. All data were analyzed using Statistical Product and Service Solutions (SPSS) version 18.0 (IBM, Armonk, NY), with GraphPad Prism for graphic presentation.

## Results

### APG-2449 is an ALK/ROS1/FAK triple-kinase inhibitor

**Fig. 1:**
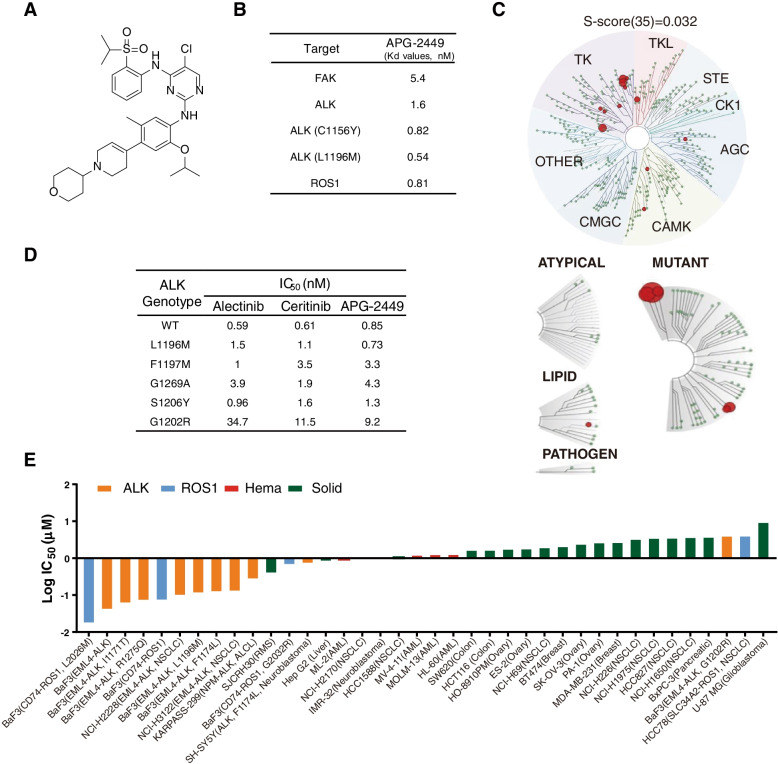
APG-2449 exhibits potent inhibitory activity against ALK/ROS1/FAK kinases and proliferation of relevant cell lines. **A**, Chemical structure of APG-2449. **B**, Dissociation constants (Kd values, nM) of APG-2449 calculated from KINOMEscan®. **C**, TREEspot interaction map of KINOMEscan selectivity panel for APG-2449 at 100 nM. Red spheres: kinases inhibited to < 35% of controls. Circle size reflects binding affinity. **D**, Inhibition of wild-type (*wt*) *ALK* and *ALK* mutants by APG-2449 and second-generation ALK inhibitors alectinib and ceritinib, using LANCE TR-FRET assay (IC_50_). **E**, Antiproliferative activity of APG-2449 (log IC_50_) in a panel of cancer cell lines. Data represent the mean of at least 2 independent experiments performed with triplicates. LANCE TR-FRET, lanthanide chelate excite time-resolved fluorescence resonance energy transfer

Profiling against 468 kinases revealed that APG-2449 (structure shown in Fig. 1A) inhibits ALK, ROS1, and FAK with Kd values of 1.6, 0.81, and 5.4 nM, respectively (Fig. 1B and C).

APG-2449 also inhibited other kinases, including insulin-like growth factor 1 receptor (IGF1R) (9 nM), leucine-rich repeat kinase 2 (LRRK2; 6.5 nM), and leukocyte receptor tyrosine kinase (LTK; 0.62 nM). APG-2449 exhibited nanomolar potency against *wt*-*ALK* and *ALK* with resistant mutations; IC_50_ values ranging from 0.85 to 9.2 nM in the LANCE TR-FRET assay with APG-2449 were similar to values for 2G ALK inhibitors ceritinib and alectinib (Fig. 1D and Suppl. Fig. 1).

Cells harboring *ALK* or *ROS1* rearrangements and other clinically relevant resistant mutations were among the most sensitive to APG-2449 (Fig. 1E). In cells without *ALK* or *ROS1* rearrangements but with detectable expression of FAK protein, APG-2449 exhibited moderate antiproliferative activity (e.g., IC_50_ = 3.55 μM in NCI-H1975 and 2.71 μM in PA-1). In conclusion, APG-2449 is a novel and potent multikinase inhibitor with selective activity against ALK, ROS1, and FAK.

### Antitumor activity of APG-2449 in *ALK*^*+*^ or *ROS1*^*+*^ murine xenograft tumor models

**Fig. 2:**
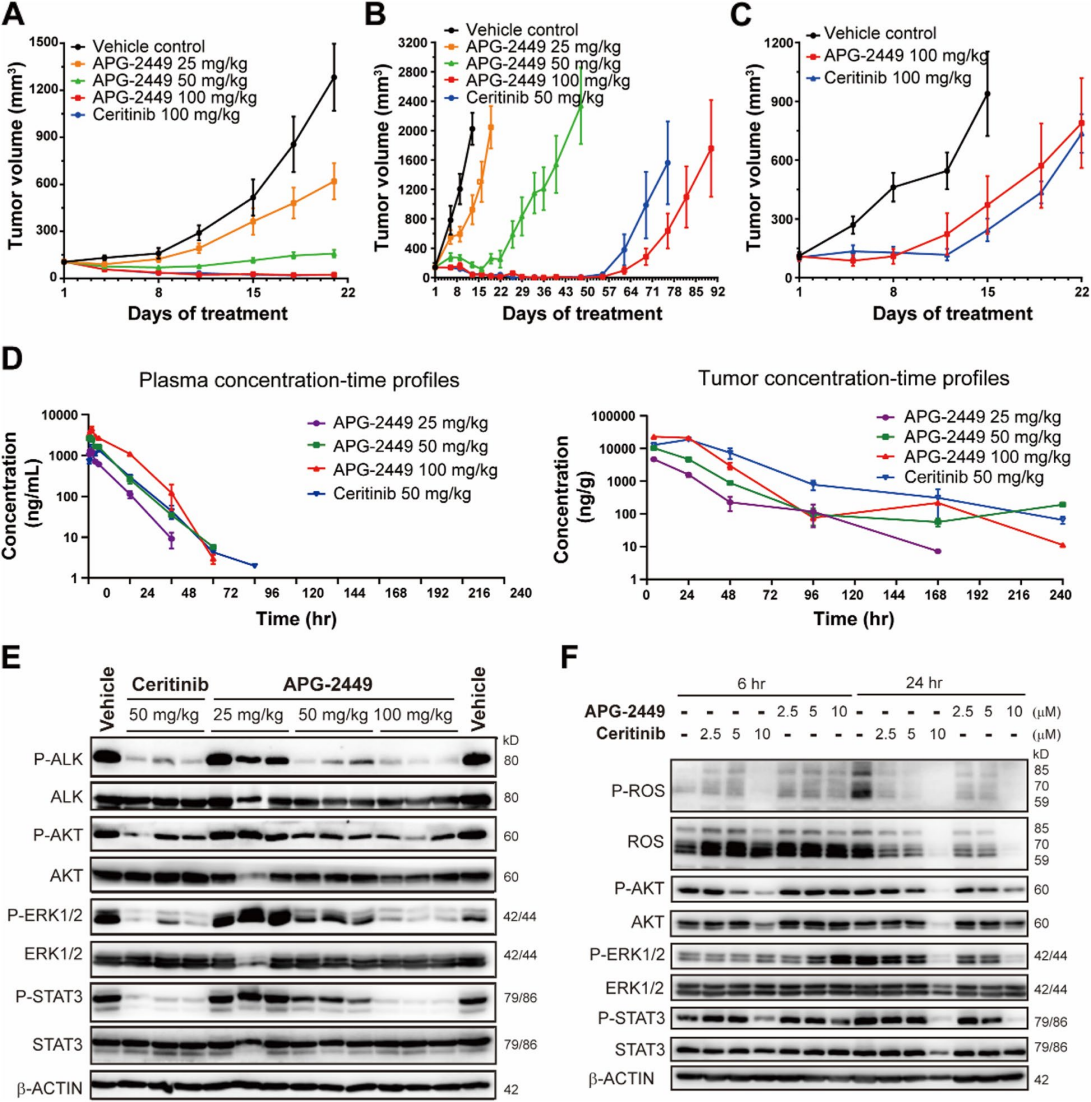
APG-2449 demonstrates antitumor activity and pharmacokinetic/pharmacodynamic correlation in *ALK*- or *ROS1*-positive xenografts in vivo. Antitumor activity of APG-2449 was evaluated in mice bearing subcutaneous tumors derived from H3122 NSCLC cells carrying *EML4-ALK* fusion gene (**A**; treated for 3 weeks, *n* = 7 per treatment group; T/C values were assessed on Day 21), KARPAS-299 cells carrying *NPM-ALK* fusion gene (**B**; treated for 3 weeks, *n* = 7 per treatment group; T/C values were assessed on Day 13), or Ba/F3 cells carrying *CD74-ROS1* fusion gene (**C**; the control group was treated for 2 weeks and the other groups for 3 weeks, *n* = 5 per treatment group; T/C values were assessed on Day 15). **D**, Pharmacokinetics of APG-2449 in plasma (*left panel*) or tumors (*right panel*) of mice carrying KARPAS-299-derived CDX tissues (treated once, *n* = 3 per treatment group). **E**, Western blot analysis of ALK signaling pathway in tumors collected 24 hours after administration from the same experiment as shown in (**D**). Each lane represents a tumor collected from an individual animal. **F**, Western blot analysis of ROS1 signaling pathway in HCC78 cells carrying *SLC34A2-ROS1* fusion following treatments in vitro. Dimethyl sulfoxide (DMSO) was included as a control. EML4, echinoderm microtubule-associated protein-like 4; NPM, nucleophosmin; SLC34A2, solute carrier family 34 member 2

In nude mice bearing NSCLC H3122 CDX tumors with *EML4-ALK* fusion, APG-2449 25, 50, or 100 mg/kg exerted dose-dependent antitumor activity, with T/C (%) values of 57.8% (1/7 stable disease [mSD], 6/7 progressive disease [mPD]), 17.5% [1/7 partial response [mPR], 6/7 mSD), and 5% (1/7 complete response [mCR], 6/7 mPR), respectively (Fig. 2A). Treatment with ceritinib 100 mg/kg was associated with a T/C (%) value of 2.3% (3/7 mCR, 4/7 mPR). No animal had severe body weight loss (Suppl. Fig. 2A).

In severe combined immunodeficient (SCID) mice bearing KARPAS-299 CDX tumors with nucleophosmin-ALK (*NPM-ALK*) fusion, APG-2449 25, 50, or 100 mg/kg demonstrated antitumor activity, with T/C (%) values of 48% (7/7 mPD), 5% (4/7 mSD), and 2% (7/7 mCR), respectively. Treatment with ceritinib 50 mg/kg resulted in a T/C (%) of 2% (7/7 mCR) (Fig. 2B). In nude mice bearing xenograft tumors derived from Ba/F3 cells with CD74-ROS1 fusion, treatment with APG-2449 or ceritinib 100 mg/kg was associated with a T/C (%) value of 37.7% or 41.6%, respectively (Fig. 2C). These findings suggest that APG-2449 exerts potent antitumor activity in *ALK*^*+*^ and *ROS*^*+*^ tumor models, with antitumor activity comparable to that of ceritinib.

### Pharmacologic profiles of APG-2449

To explore pharmacologic characteristics of APG-2449, we treated SCID-beige mice bearing KARPAS-299 CDX tumors with various single oral doses of APG-2449, which resulted in dose-proportional increases in APG-2449 plasma and tumor exposures (Fig. 2D).

Twenty-four hours after administration, APG-2449 exerted dose-dependent suppression of tumor phosphorylated ALK (p-ALK), Ak strain transforming (p-AKT), extracellular signal-regulated kinase (p-ERK1/2), and signal transducer and activator of transcription (p-STAT3; Fig. 2E). Similarly, treatment of HCC78 cells with solute carrier family 34-member 2 (*SLC34A2-ROS1*) fusion with APG-2449 induced downregulation of p-ROS1, p-AKT, p-ERK1/2, and p-STAT3 after 24 h (Fig. 2F). Collectively, these results suggest that certain antitumor effects of APG-2449 are mediated by suppressing ALK- or ROS1-driven signaling pathways.

### APG-2449 exerts potent and durable in vivo antitumor activity against acquired (secondary) *ALK*- and *ROS1*-resistant mutations

**Fig. 3:**
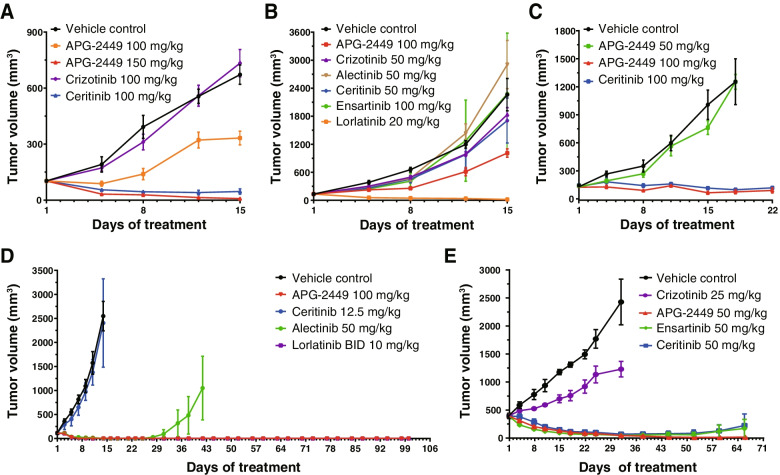
Secondary *ALK* or *ROS1* mutations conferring drug resistance are responsive to APG-2449 in vivo. Antitumor activity of APG-2449 against acquired, drug-resistant mutations was evaluated in subcutaneous xenograft models derived from Ba/F3 cells carrying *EML4-ALK*^*L1196M*^ mutation (**A**; treated for 2 weeks, *n* = 6 per treatment group), Ba/F3 cells carrying *EML4-ALK*^*G1202R*^ mutation (**B**; treated for 2 weeks, *n* = 5 per treatment group), Ba/F3 cells carrying *CD74-ROS1*^*L2026M*^ mutation (**C**; treated for 3 weeks while 2 groups were terminated earlier as indicated, *n* = 4 or 5 per treatment group), NSCLC PDX LD1–0006-390,637 harboring *ALK*^*L1196M_G1202R*^ double mutations (**D**; treated for 3 weeks, *n* = 5 per treatment group), or NSCLC PDX LU-01-0582R with acquired resistance to crizotinib (**E**; treated for 4 weeks, *n* = 3 or 4 per treatment group)

Clinical resistance to ALK and ROS1 inhibitors frequently develops because of acquired resistance mutations, including *ALK*^*G1202R*^, *ROS1*^*L2026M*^, and *ALK*^*L1196M*^, the last of which is the most common crizotinib-resistant mutation. Nude mice bearing *ALK*^*L1196M*^-mutant tumors were resistant to treatment with crizotinib 100 mg/kg (Fig. 3A). In contrast, APG-2449 100 and 150 mg/kg dose dependently suppressed tumor growth. Paralleling these findings, ceritinib 100 mg/kg exhibited substantial antitumor activity, with an ORR of 83% (1/6 mCR, 4/6 mPRs, and 1/6 mSD). At the same dose, APG-2449 appeared to be more potent than ceritinib, with an ORR of 100% (4/6 mCRs, 2/6 mPRs).

*ALK*^*G1202R*^ is the most frequently observed mutation that is resistant to 2G ALK inhibitors [27, 28]. In SCID mice with *ALK*^*G1202R*^-mutant tumors, APG-2449 demonstrated stronger antitumor activity on Day 15 (T/C % value of 44.54%) than crizotinib (84.3%), alectinib (130%), ceritinib (76.7%), or ensartinib (97.9%). In these difficult-to-treat *ALK*^*G1202R*^-mutant tumors, only the recently approved 3G ALK inhibitor lorlatinib showed marked activity (T/C % value of 0.8%) (Fig. 3B). In mice bearing *ROS1*^*L2026M*^-mutant tumors, APG-2449 demonstrated potent antitumor activity, with 2/5 mPR and 3/5 mSD compared to 4/4 mSD in the ceritinib-treated group (Fig. 3C).

NSCLC PDX models were further employed to investigate the effect of APG-2449 on ALK inhibitor-resistant mutations. In NU/NU mice bearing LD1–0006-390,637 tumors harboring concurrent *ALK*^*L1196M_G1202R*^ mutations, APG-2449 demonstrated marked antitumor activity and achieved 100% mCR (5/5 mice) as early as Day 11 of treatment (effects similar to those with lorlatinib; Fig. 3D). Alectinib also achieved 100% mCR on Day 16 of treatment, but relapse was observed on Day 28. In another independent experiment using this model, treatment with APG-2449, alectinib, or lorlatinib achieved 100% mCR that was sustained for at least 3 weeks (Suppl. Fig. 2B). Disease progression was observed in the lorlatinib group on Day 39, whereas APG-2449- and alectinib-treated tumors remained unpalpable. On the other hand, BALB/c nude mice bearing crizotinib-resistant LU-01-0582R tumors remained resistant to crizotinib 25 mg/kg (Fig. 3E), while treatment with APG-2449, ceritinib, or ensartinib demonstrated potent antitumor activity, resulting in 100, 75, and 80% mCR, respectively (Fig. 3E). Disease progression occurred in the ceritinib and ensartinib groups on Day 59, whereas APG-2449 sustained 100% mCR up to Day 66.

In conclusion, our in vivo studies demonstrated potent and durable activity of APG-2449 against tumors with *ALK* or *ROS1* secondary mutations, including the critical *ALK*^*G1202R*^. Compared to ceritinib or ensartinib, APG-2449 exhibits greater antitumor activity against crizotinib-resistant tumors lacking secondary *ALK* mutations, indicating that APG-2449 may be effective in a large proportion of patients with ALK inhibitor-resistant NSCLC irrespective of *ALK* genotype.

### APG-2449 sensitizes ovarian cancer to chemotherapy by inhibiting the FAK signaling pathway

**Fig. 4:**
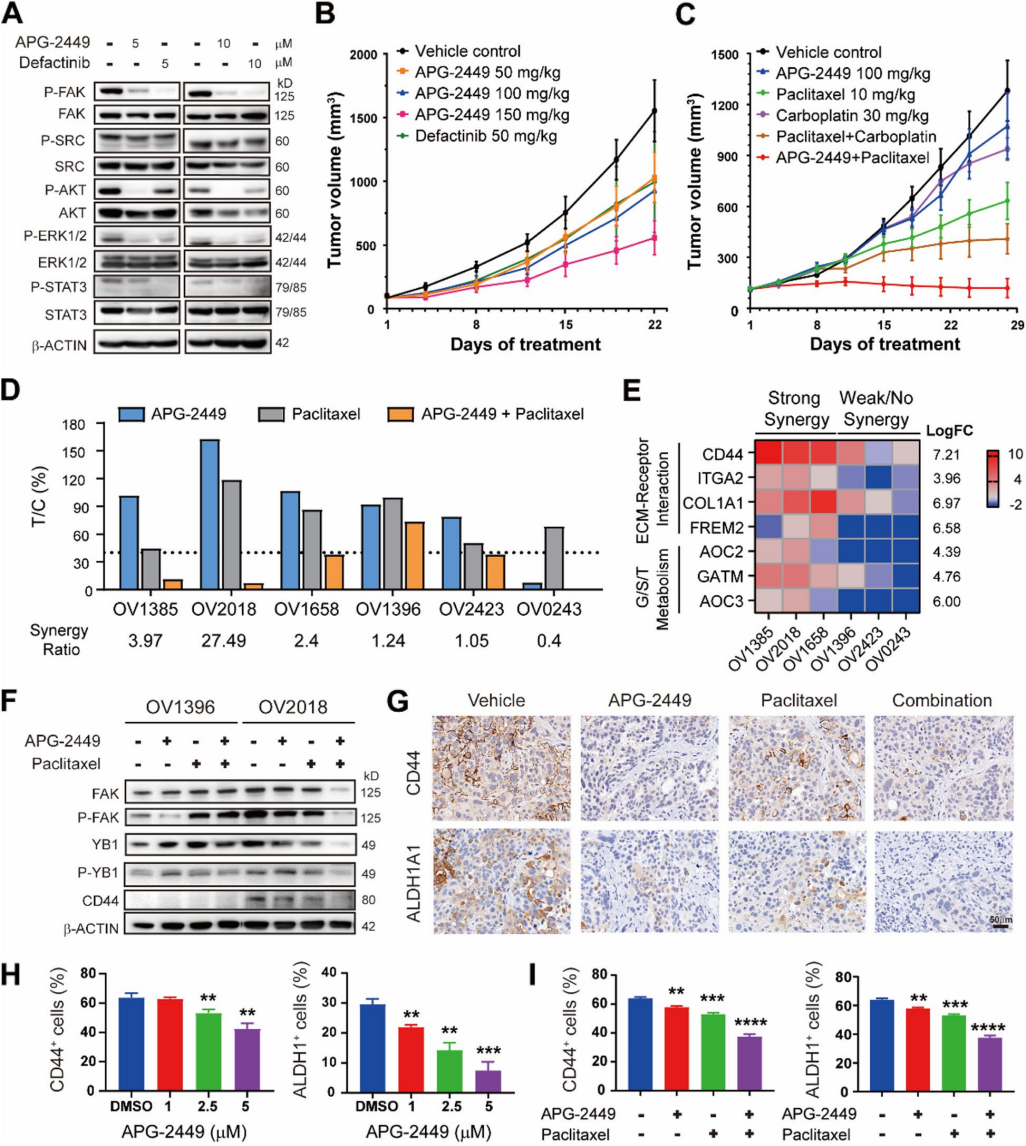
Combination of APG-2449 and paclitaxel inhibits tumor growth of FAK-expressing ovarian cancer xenografts in mice. Western blotting of FAK signaling pathway in PA-1 ovarian cells treated with APG-2449 or defactinib for 4 hours in vitro (**A**). Efficacy studies in subcutaneous CDX models derived from PA-1 (**B**; treated for 3 weeks, *n* = 5 per treatment group) or OVCAR-3 (**C**; treated for 4 weeks, *n* = 5 per treatment group; T/C values assessed on Day 28) ovarian cancer cells. **D**, A mouse trial experiment was conducted in a panel of 6 ovarian cancer PDX models (treated for 3–6 weeks, *n* = 2 per treatment group). **E**, Heatmap of genes significantly changed in ovarian PDX tumors that responded to APG-2449 plus paclitaxel combination (synergy ratio > 2) versus nonresponders (synergy ratio < 2). *p* < 0.05 (false discovery rate < 0.05). **F**, Western blot analysis of FAK signaling pathways in tumor samples responsive (OV2018) and nonresponsive (OV1396) to APG-2449 plus paclitaxel treatment; tumor samples collected 4 hours after the last administration from the experimental animals shown in panel **D**. **G**, IHC staining of CD44- or ALDH1A1-positive cells in OVCAR-3 tumors collected 4 hours after the last treatment from tumor-bearing mice treated with APG-2449, paclitaxel or the combination for 10 days. **H**, SKOV-3 cells were treated with increasing doses of APG-2449 for 72 hours. **I**, SKOV-3 cells were treated with APG-2449 (1 μM), paclitaxel (5 nM), or the combination for 72 hours. CD44^+^ and ALDH1A^+^ cells were determined by flow cytometry. ***p < 0.001, **p < 0.01 vs. DMSO controls. Date presented are representative of 2 independent experiments as the mean ± SEM of triplicate biological replicates

Overexpression and amplification of *FAK* occurs in 68 and 26.7% of patients with ovarian cancer, respectively [29]. These genetic alterations are significantly associated with metastasis and a poor prognosis [30]. Therefore, the FAK signaling pathway represents a novel avenue for pharmacologic intervention against ovarian cancer. We thus evaluated on-target activity of APG-2449 against FAK in preclinical models of ovarian cancer. Immunoblotting analysis revealed that APG-2449 downregulated Y397-FAK autophosphorylation (p-FAK) and downstream signaling factors p-AKT, p-ERK1/2, and p-STAT3 in PA-1 ovarian cancer cells (Fig. 4A). Interestingly, the inhibitory effect of APG-2449 on p-AKT was more potent than that of FAK-selective inhibitor defactinib. However, defactinib inhibited p-FAK to a greater extent than APG-2449, suggesting that the 2 agents have distinct pharmacologic properties.

We next evaluated the antitumor activity of APG-2449, alone or combined with standard-of-care (SOC) chemotherapeutics, in ovarian cancer xenograft models. In SCID mice bearing PA-1 CDX tumors, APG-2449 50, 100, or 150 mg/kg showed dose-dependent antitumor activity (Fig. 4B) and downregulation of p-FAK (Suppl. Fig. 3A). Conversely, APG-2449, carboplatin, or paclitaxel monotherapy showed weak or marginal antitumor activity in BALB/c nude mice bearing ovarian cancer OVCAR-3 CDX tumors, (Fig. 4C). The SOC combination of carboplatin plus paclitaxel suppressed tumor growth, with a T/C value of 33.9%. Of potential interest, APG-2449 combined with paclitaxel had potent antitumor activity, with a T/C value of 8.2% and DCR of 80% (i.e., 1/5 mPR, 3/5 mSD). Ternary combinations of APG-2449, paclitaxel, and carboplatin did not further improve antitumor activity (data not shown). All animals were tolerant of the combinations, with limited body weight loss and no interruptions in dosing (Suppl. Fig. 3B).

To confirm the therapeutic potential of APG-2449 in combination with paclitaxel, we conducted an experiment in murine ovarian cancer PDX models. From a group of 78 ovarian cancer PDXs, we selected 6 exhibiting the highest expression levels of *FAK* mRNA or *FAK* amplification according to RNA-seq (Suppl. Table S2). Compared to each single agent, the combination of APG-2449 and paclitaxel enhanced antitumor activity in all 6 PDX models (Fig. 4D). In addition, the combination significantly augmented antitumor activity in 3 of the 6 PDX models, with a synergy ratio greater than 2 (Fig. 4D). Interestingly, these 3 responsive models were not sensitive to carboplatin treatment (Suppl. Table S2). In the aggregate, our studies demonstrate potent activity of APG-2449 plus paclitaxel in preclinical models of ovarian cancer exhibiting *FAK* overexpression or amplification, including those insensitive to carboplatin.

### Reduction of ovarian Cancer Stem Cell (CSCs) by APG-2449 and paclitaxel

To explore the mechanism of action underlying enhanced and/or synergistic antitumor effects of APG-2449 plus paclitaxel in ovarian cancer, we compared gene expression profiles of untreated PDX tumors obtained from responders (synergy ≥2) and nonresponders (synergy < 2) according to RNA-seq data. Genes upregulated in responders were identified as an extracellular matrix (ECM)-receptor interaction set (CD44, integrin subunit α 2 [ITGA2], collagen type I α 1 [COL1A1], FRAS1-related ECM [FREM2]) and a glycine, serine, threonine metabolism set (amine oxidase copper containing [AOC2, AOC3], glycine amidinotransferase, mitochondrial [GATM]) (Fig. 4E). ECM is a structural component of the tumor microenvironment that is known to support proliferation, self-renewal, and differentiation of CSCs [31]. Upregulation of the glycine, serine, threonine pathway supports tumor homeostasis and promotes cancer cell survival [32]. Thought-provokingly, the most markedly upregulated gene in APG-2449 responders was CD44, which is a marker for CSCs in ovarian cancer [33]. IHC staining further confirmed that CD44 expression was significantly higher in responders (vs. nonresponders), whereas no such differences were observed for FAK, P-FAK or E-cadherin (Suppl. Fig. 3C and D). These results suggest that high-“stemness” ovarian cancer models may be more susceptible to APG-2449/paclitaxel treatment.

In ovarian cancer models, it has been reported that FAK inhibition reduces CD44 protein expression levels [34] and stem phenotype [35]. In our CD44-expressing OV2018 PDX model that responded to APG-2449/paclitaxel (Fig. 4D), this binary combination downregulated CD44 protein expression (Fig. 4F). Downregulation of FAK, p-FAK, YB1, and p-YB1 was also noted, suggesting that downregulation of CD44 may be mediated by its upstream transcription factor YB1. In contrast, CD44 expression levels were barely detectable in OV1396 tumors insensitive to the combination. IHC analysis of OVCAR-3 tumors consistently revealed that expression of CD44 and another CSC marker (ALDH1A1) decreased after APG-2449/paclitaxel treatment (Fig. 4G). To assess effects on numbers of CSCs, we exposed SKOV-3 cells to APG-2449, paclitaxel, and the combination, and then analyzed by flow cytometry, which showed that APG-2449 dose dependently decreased numbers of CD44^+^ and ALDH1^+^ CSCs (Fig. 4H). A further decrease in these CSC populations was observed when APG-2449 was combined with paclitaxel (Fig. 4I). These results demonstrate that the combination of APG-2449 and paclitaxel effectively inhibits tumor growth in ovarian cancers with *FAK* overexpression or amplification by downregulating CD44^+^ and ALDH^+^ CSC populations.

### APG-2449 enhances EGFR TKI-mediated tumor suppression in NSCLC

**Fig. 5:**
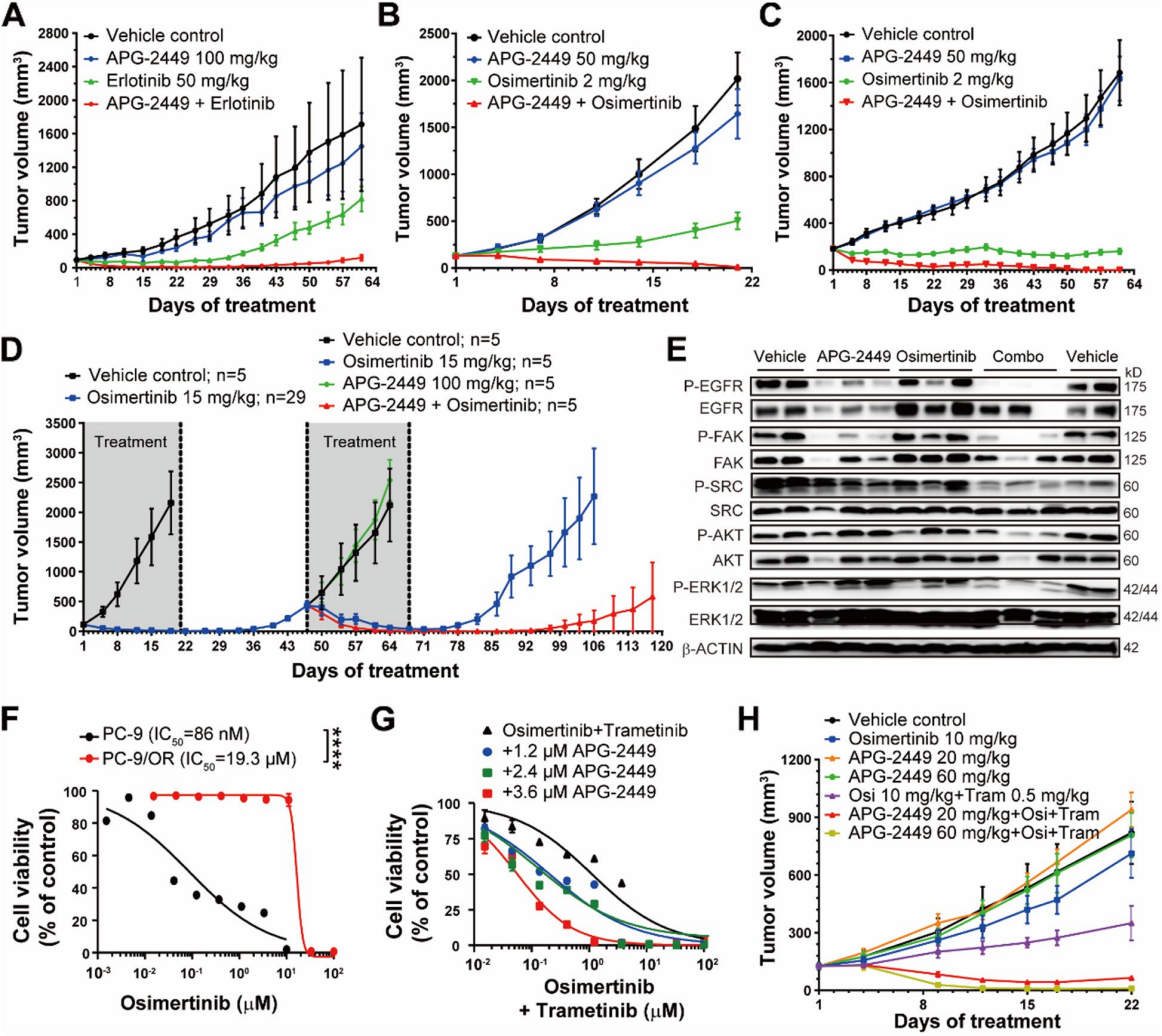
APG-2449 synergizes with EGFR inhibitors and overcomes osimertinib-resistance when combined with osimertinib/trametinib in xenograft models. Combination treatments with APG-2449 and EGFR inhibitors were evaluated in an HCC827 CDX model (**A**; treated for 3 weeks, *n* = 5 per treatment group), NCI-H1975 CDX model (**B**; treated for 3 weeks, *n* = 5 per treatment group), and NSCLC PDX model LD1–0006-215,676 harboring *EGFR*^*L858R_T790M*^ and *ROS1* fusion (**C**; treated for 61 days, *n* = 6 per treatment group) in mice. **D**, Tumor growth curve of NCI-H1975 CDX model rechallenged with APG-2449 plus osimertinib (Day 47- Day 67) upon disease progression from 21-day osimertinib treatment (Day 1-Day 21). **E**, Western blot analysis of EGFR and FAK downstream signaling in NCI-H1975 xenografts collected 4 hours after the last administration from tumor-bearing mice treated with indicated compounds for 2 weeks. Each lane represents an individual animal. **F**, Cell viability (IC_50_) of PC-9 (parental) or PC-9/OR (osimertinib-resistant) cells treated with osimertinib at indicated concentrations for 3 days. Data are representative of 3 independent experiments and shown as the mean ± SEM of triplicates. ^****^p < 0.0001. **G**, Cell viability of PC-9/OR cells treated with indicated compounds for 72 hours. **H**, PC-9/OR xenograft models were treated with the indicated compounds for 3 weeks (*n* = 5 per treatment group)

FAK and SRC family kinases (SFK) support downstream AKT and mitogen-activated protein kinase (MAPK) signaling when continuous EGFR inhibition is driven by EGFR TKIs [20, 36]. Incomplete inhibition of these survival signals can gradually contribute to EGFR TKI-acquired resistance. To enhance antitumor activity and delay relapse, combination therapy with FAK/SRC inhibitors may be a viable approach. We investigated the effect of combined APG-2449 and EGFR TKIs in mice bearing xenografts derived from (1) NSCLC HCC827 with *EGFR*^*Ex19del*^ (SCID-beige mice); (2) NCI-H1975 with *EGFR*^*L858R_T790M*^ mutation (BALB/c nude mice); and (3) a PDX model with *EGFR*^*L858R_T790M*^ and *ROS1* fusion (LD1–0006-215,676; NU/NU mice). A strong synergistic antitumor effect was observed in the HCC827 CDX model when APG-2449 was combined with first-generation EGFR inhibitor erlotinib (Fig. 5A). A similar effect was observed when APG-2449 was combined with 3G EGFR inhibitor osimertinib (2 mg/kg) in both a CDX (NCI-H1975; Fig. 5B) and a PDX (LD1–0006-215,676; Fig. 5C) model. The combination treatment resulted in 100% ORRs across all models tested. Administration of APG-2449 in concert with a higher dose of osimertinib (15 mg/kg) extended the duration of antitumor response, with a temporary loss of body weight (Suppl. Fig. 4A and B).

We next evaluated antitumor activity of APG-2449 plus osimertinib in an osimertinib-relapsed setting using the NCI-H1975 CDX model in BALB/c nude mice. Tumor-bearing mice were initially treated with a high dose of osimertinib (15 mg/kg) for 21 days, after which all treated animals achieved complete response. Treatment was then suspended from Day 22 until disease progression. Mice carrying relapsed tumors were randomly allocated to treatment with APG-2449, alone or combined with osimertinib, for 21 days. Rechallenge with APG-2449 resensitized relapsed tumors to osimertinib (1/5 mCR), with gradual disease progression after dose suspension. Intriguingly, treatment with APG-2449 plus osimertinib achieved deeper and more durable response (5/5 mCR) compared to osimertinib alone (Fig. 5D).

As a putative mechanism, we postulate that combined treatment with APG-2449 and osimertinib suppressed phosphorylation of EGFR (p-EGFR), FAK (p-FAK), SRC (p-SRC), and ERK (p-ERK) compared to each single agent, in NCI-H1975 xenografts after 2 weeks (Fig. 5E, Suppl. Fig. 4C). In summary, the combination of APG-2449 and EGFR TKIs synergistically enhances antitumor activity in *EGFR*-mutant NSCLC models, with synergy driven by downregulation of FAK, EGFR, SRC, and ERK phosphorylation and APG-2449 extending the duration of response to osimertinib.

To determine whether the combination of APG-2449, EGFR TKIs, and MEK TKIs can overcome acquired resistance to osimertinib in *EGFR*-mutated NSCLC, we analyzed the antiproliferative activity of APG-2449, alone or in tandem with other agents, in PC-9/OR cells with acquired osimertinib resistance (Fig. 5F). Although the combination of APG-2449 and osimertinib did not inhibit cellular proliferation (data not shown), adding MEK inhibitor trametinib reduced proliferation of PC-9/OR cells (Fig. 5G). In BALB/c nude mice bearing osimertinib-resistant PC-9/OR CDX xenografts, (1) treatment with APG-2449 or osimertinib alone exerted negligible antitumor activity, (2) osimertinib plus trametinib had moderate effects, and (3) the triad of osimertinib, trametinib, and APG-2449 (20 mg/kg or 60 mg/kg) demonstrated potent antitumor activity (Fig. 5H), with an ORR of 100% (5/5 mPRs with APG-2449 20 mg/kg or 1/5 mCR and 4/5 mPR with APG-2449 60 mg/kg). These results indicate that treatment with APG-2449, in tandem with a 3G EGFR inhibitor and a 3G MEK inhibitor, can overcome osimertinib resistance.

## Discussion

Targeting oncogenic-driving genetic aberrations using small-molecule drugs has met with considerable clinical successes and is a viable strategy of pharmacologic innovation against a range of malignancies. Among these druggable targets, *ALK* and *ROS1* rearrangements, as well as *EGFR* mutations in NSCLC, are well-known examples. Despite high initial response rates, patients almost inevitably develop TKI resistance via multiple disease mechanisms, including: (1) acquiring de novo resistance mutations; (2) expanding pre-existing resistance mutations; (3) triggering gene amplification; and/or (4) activating alternative survival signaling pathways [37]. *FAK* overexpression or amplification has been linked to tumor progression, metastasis, drug resistance, and a poor prognosis in patients with various solid tumors [38]. Consequently, extensive efforts have been undertaken to: (1) develop next-generation TKIs that overcome clinically relevant resistant mutations and (2) discover novel combination therapies that suppress resistance mechanisms.

**Fig. 6:**
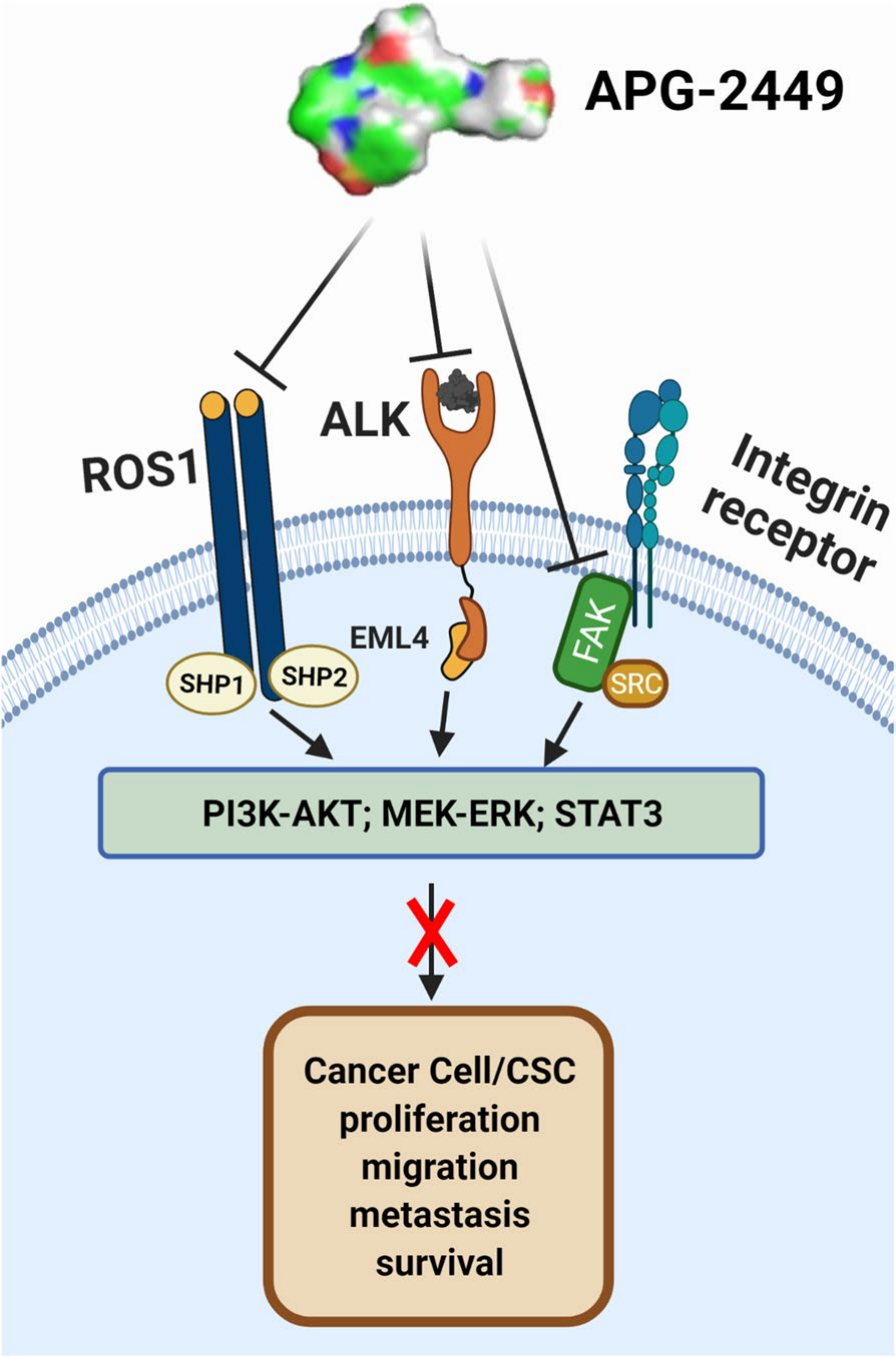
Scheme for the mechanisms of action of APG-2449. Created with BioRender (Toronto, Ontario, Canada). CSC, cancer stem cell; EML, echinoderm microtubule-associated protein-like; MEK-ERK, mitogen-activated protein kinase kinase-extracellular signal-regulated kinase; PI3K-AKT, phosphatidylinositol 3-kinase and protein kinase B; SHP, SH2 containing protein tyrosine phosphatase; STAT, signal transducer and activator of transcription

Our report suggests that APG-2449 may help to meet these needs by serving as (1) a novel ALK/ROS1 inhibitor that can overcome acquired drug resistance conferred by secondary mutations and other resistance mechanisms in *ALK*^*+*^*/ROS1*^*+*^ NSCLC; (2) a unique FAK inhibitor with a potentially different pharmacologic profile; and (3) an important constituent of combinations with SOC chemotherapeutics or targeted agents that can reverse primary or acquired drug resistance in *FAK*-overexpressing or amplified ovarian cancer and *EGFR*-mutant NSCLC models. As a putative mechanism of action, we propose that APG-2449 induces downregulation of p-ALK, p-ROS1, p-FAK, p-AKT, p-ERK1/2, and p-STAT3, hence suggesting that its antitumor activity is mediated by inhibiting multiple salient oncogenic pathways (Fig. 6).

Secondary mutations confer acquired resistance to first-generation ALK inhibitors in 20 to 30%, and 2G ALK inhibitors in 50 to 70%, of patients with *ALK*^*+*^ NSCLC [28]. Another 20 to 70% of patients develop acquired resistance without secondary mutations [28]. In our preclinical NSCLC models, APG-2449 proved to be active against critical secondary mutations *ALK*^*L1196M*^ and *ALK*^*G1202R*^ appearing independently or concurrently and exerts antitumor activity in a crizotinib-resistant NSCLC PDX lacking secondary mutations.

If confirmed in clinical trials, these findings may support APG-2449 as a member of a new generation of ALK multikinase inhibitors that can offset resistance in certain subpopulations of patients with NSCLC that relapses on ALK inhibitors. Based on our preclinical data in this setting, APG-2449 demonstrated enhanced activity compared to current ALK inhibitors, including ceritinib, ensartinib, and alectinib.

Ovarian epithelial cancer is one of the deadliest malignancies in women, up to 70% of whom have *FAK* overexpression, amplification, or activation [29], which is in turn significantly associated with higher tumor stage, metastasis, and shorter overall survival. These effects may be ascribed to the pivotal role of *FAK* in regulating cellular migration and survival, growth factor signaling, cell cycle progression, and chemoresistance [10]. Inhibition of FAK is therefore a promising pharmaceutical approach to treat ovarian cancer and/or advance its SOC (including chemotherapy). In this context, FAK inhibition by defactinib is known to resensitize ovarian tumors to paclitaxel in both preclinical and clinical settings [14, 34]. FAK inhibition by defactinib also targets the CD44^+^ CSC population and restores sensitivity in chemotherapy-resistant cells [34]. Although APG-2449 alone effectively suppresses phosphorylation of FAK in ovarian PA-1 cells, it fails to exert meaningful antitumor activity in PA-1 and OVCAR-3 ovarian cancer xenograft tumor models. This observation comports with the prevailing understanding that monotherapy with a FAK inhibitor may be insufficient in certain clinical settings [11].

On the other hand, our studies have demonstrated that paclitaxel combined with APG-2449 is more effective in inhibiting tumor growth compared to paclitaxel plus carboplatin in a carboplatin-insensitive OVCAR-3 ovarian cancer xenograft model. Platinum/taxane-based chemotherapy remains the backbone of ovarian cancer treatment, and previous studies have shown that elevated FAK activation underlies intrinsic and acquired resistance to chemotherapy in residual tumors of patient-derived cells or mouse xenograft tumors treated with platinum or taxanes [35]. FAK inhibition by defactinib is known to enhance chemosensitivity in taxane-resistant cells [34]. Confirmation of the effectiveness of APG-2449/paclitaxel in 3 of 6 ovarian cancer PDX models supports the potential advantage of this combination over paclitaxel alone. These 6 PDX models expressed high levels of *FAK* mRNA and *FAK* amplification. Among these 6 PDX models, 3 that were sensitive to combined therapy with APG-2449 and paclitaxel had high levels of CD44 expression, suggesting that *FAK* alterations and CD44 expression warrant further clinical investigation as predictive biomarkers.

Also worthy of further clinical exploration are expression levels of CD44, ALDH1, p-FAK/FAK, and p-YB1/YB1, as well as CSCs and other potential predictors and/or pharmacodynamic biomarkers, in ovarian cancer. CSCs constitute a subgroup of cancer cells responsible for primary tumor growth, metastasis, chemoresistance, and cancer relapse [39–41]. In addition to driving tumor progression and metastasis in ovarian cancer, CSCs play a critical role in conferring chemoresistance, which in turn leads to relapsed or refractory tumors [40, 42, 43]. FAK is required to maintain ovarian cancer CSCs, potentially by activating the β-catenin pathway [35, 44]. FAK overexpression upregulates ALDH1 activity in platinum-resistant (and CD44 activity in taxane-resistant) ovarian tumors [10, 34]. Expression of both ALDH1 and CD44 is associated with CSC populations in ovarian cancer [45]. In our preclinical models of ovarian cancer, tumors with high CD44 expression were more likely to respond to APG-2449/paclitaxel, and the combination also significantly reduced CSC populations expressing either ALDH1 or CD44. Collectively, synergistic antitumor activity of APG-2449/paclitaxel is likely mediated by downregulation of ALDH1^+^, CD44^+^, and CSC populations.

In lung cancer, SFK and FAK sustain AKT and MAPK pathway signaling under continuous EGFR inhibition [20]. Inhibiting either the AKT or MAPK pathway enhances the efficacy of osimertinib [20]. Therefore, the combination of EGFR or SFK/FAK inhibitors constitutes a promising therapeutic strategy for *EGFR*-mutant lung cancer. Accordingly, increased phosphorylation of FAK has been observed in erlotinib-resistant NSCLC cells [46]. Defactinib combined with osimertinib enhanced antitumor activity in PC-9- and PC-9-pemetrexed-resistant xenografts [46]. In vitro, defactinib and osimertinib also overcome erlotinib resistance [46]. However, the effects of concomitant defactinib and osimertinib in overcoming osimertinib resistance has not been evaluated. Our preclinical studies demonstrate that combining APG-2449 with osimertinib significantly enhanced antitumor activity in *EGFR*^*Ex19del*^ HCC827, *EGFR*^*L858R_T790M*^ NCI-H1975, and *EGFR*^*L858R_T790M*^ plus *ROS1* fusion in xenograft and PDX models. Compared to osimertinib alone, the combination with APG-2449 further suppressed phosphorylation of EGFR, SRC, AKT, and ERK in H1975 tumors.

In addition to SFK and FAK signaling cascades, cancer cells may acquire resistance to osimertinib by activating the ERK1/2 pathway [47]. Osimertinib combined with a MEK or ERK inhibitor synergistically decreased survival in *EGFR*-mutant, but not *wt-EGFR* NSCLC cells [48]. In osimertinib-resistant PC-9/OR cells and xenografts, APG-2449 plus osimertinib failed to achieve tumor regression, indicating that alternative signaling pathways may be activated to sustain tumor growth. However, the ternary combination of APG-2449, osimertinib, and MEK inhibitor trametinib achieved 100% ORR, resulting in a CR or PR in all animals.

## Conclusions

In conclusion, investigational agent APG-2449 is a novel small-molecule inhibitor targeting ALK/ROS1/FAK. In our preclinical models including APG-2449 alone or in concert with other agents, this multikinase inhibitor demonstrated anticancer activity in various types of solid tumors, including NSCLC carrying *ALK*, *ROS1*, or *EGFR* mutations and ovarian cancer expressing *FAK* alterations and elevated CD44 protein expression. Administered alone or in combination, APG-2449 can overcome primary and acquired TKI resistance in preclinical models of NSCLC and ovarian cancer. If confirmed in clinical trials, these data may pave the way for APG-2449 to become an innovative therapy for patients with certain solid tumors. Consistent with the encouraging preclinical findings, a phase 1 clinical trial has been initiated to evaluate the safety and preliminary efficacy of APG-2449 in patients with *ALK*^*+*^ NSCLC and other solid tumors (NCT03917043/CTR20190468).

## Supplementary Information

**Additional file 1.**

**Additional file 2: Figure S1.** Inhibition curves of ALK kinase activity by APG-2449 and reference compounds alectinib and ceritinib assessed by LANCE TR-FRET assay. Wild-type (*wt*) *ALK* (A), *ALK* ^*L1196M*^ mutant (B), *ALK* ^*F1197M*^ mutant (C), *ALK* ^*G1269A*^ mutant (D), *ALK* ^*S1206Y*^ mutant (E) and *ALK* ^*G1202R*^ mutant (F). LANCE TR-FRET, lanthanide chelate excite time-resolved fluorescence resonance energy transfer. **Figure S2.** Antitumor activity of APG-2449 in ALK-positive xenograft tumor models in mice. (A) Assessment of changes in body weight (%) of mice bearing H3122 xenograft tumors as shown in Fig. 2A. (B) Independent repeat experiment in NSCLC PDX LD1–0006-390,637 (treated for 3 weeks, *n* = 3–5 per treatment group) as shown in Fig. 3D. **Figure S3.** Combination of APG-2449 and paclitaxel inhibits tumor growth in ovarian cancer xenograft models in mice. (A) Western blotting analysis of FAK downstream signaling in PA-1 tumors collected from experiment as shown in Fig. 4B. (B) Changes in body weights (%) of OVCAR-3 xenograft-bearing mice as shown in Fig. 4C. IHC staining (C) and quantitation of staining intensity (D) of CD44, E-Cadherin (E-Cad), FAK, and p-FAK in untreated PDX tumors shown in Fig. 4D. **Figure S4.** Enhancement of osimertinib-mediated tumor suppression by APG-2449 in NSCLC. (A) Repeated efficacy study using a higher dose of osimertinib (15 mg/kg) in NSCLC PDX LD1–0006-215,676 (vehicle and APG-2449 groups were treated for 16 days and other groups for 23 days, *n* = 8–10 per treatment group). (B) Changes in body weights (%) of tumor-bearing mice in the experiment shown in A. (C) Protein expression levels shown in Fig. 5E were quantitated and shown as mean ± SEM relative to the loading control β-actin or total proteins where phosphorylated proteins were assessed (*n* = 3 mice per treatment group). *p < 0.05, **p < 0.01 vs. vehicle control. **Supplementary Table S1.** Antibodies used for western blotting. **Supplementary Table S2.** Genetic characteristics of ovarian cancer PDX models.

## Data Availability

The datasets generated and/or analyzed during the current study are available in the NCBI repository (PRJNA813965): https://www.ncbi.nlm.nih.gov/bioproject/PRJNA813965

## Abbreviations

AKT: Ak strain transforming protein
ALDH: Aldehyde dehydrogenase
ALK: Anaplastic lymphoma kinase
CDX: Cell-line-derived xenograft
CR: Complete Response
CSC: Cancer stem cell
DCR: Disease control rate
EGFR: Epidermal growth factor receptor
ERK: Extracellular signal-regulated kinase
FAK: Focal adhesion kinase
HRP: Horseradish Peroxidase
IHC: Immunohistochemistry
MAPK: Mitogen-activated protein kinase
MEK: Mitogen-activated extracellular signal-regulated kinase
NSCLC: Non-small-cell lung cancer
ORR: Objective response rate
PD: Progressive disease
PDX: Patient-derived xenograft
PK: Pharmacokinetics
PR: Partial response
PO: Administered orally
QD: Once daily
SCID: Severe combined immunodeficient
SD: Stable disease
SFK: SRC family kinases
SOC: Standard-of-care
SPSS: Statistical Product and Service Solutions
TKI: Tyrosine kinase inhibitors
