# Supplementary material for: Discovery of a novel ALK/ROS1/FAK inhibitor, APG-2449, in preclinical non-small cell lung cancer and ovarian cancer models
Source: BMC Cancer. 2022 Jul 11;22:752. doi: 10.1186/s12885-022-09799-4 (PMC9277925; doi:10.1186/s12885-022-09799-4)

Original images for Figure 2E

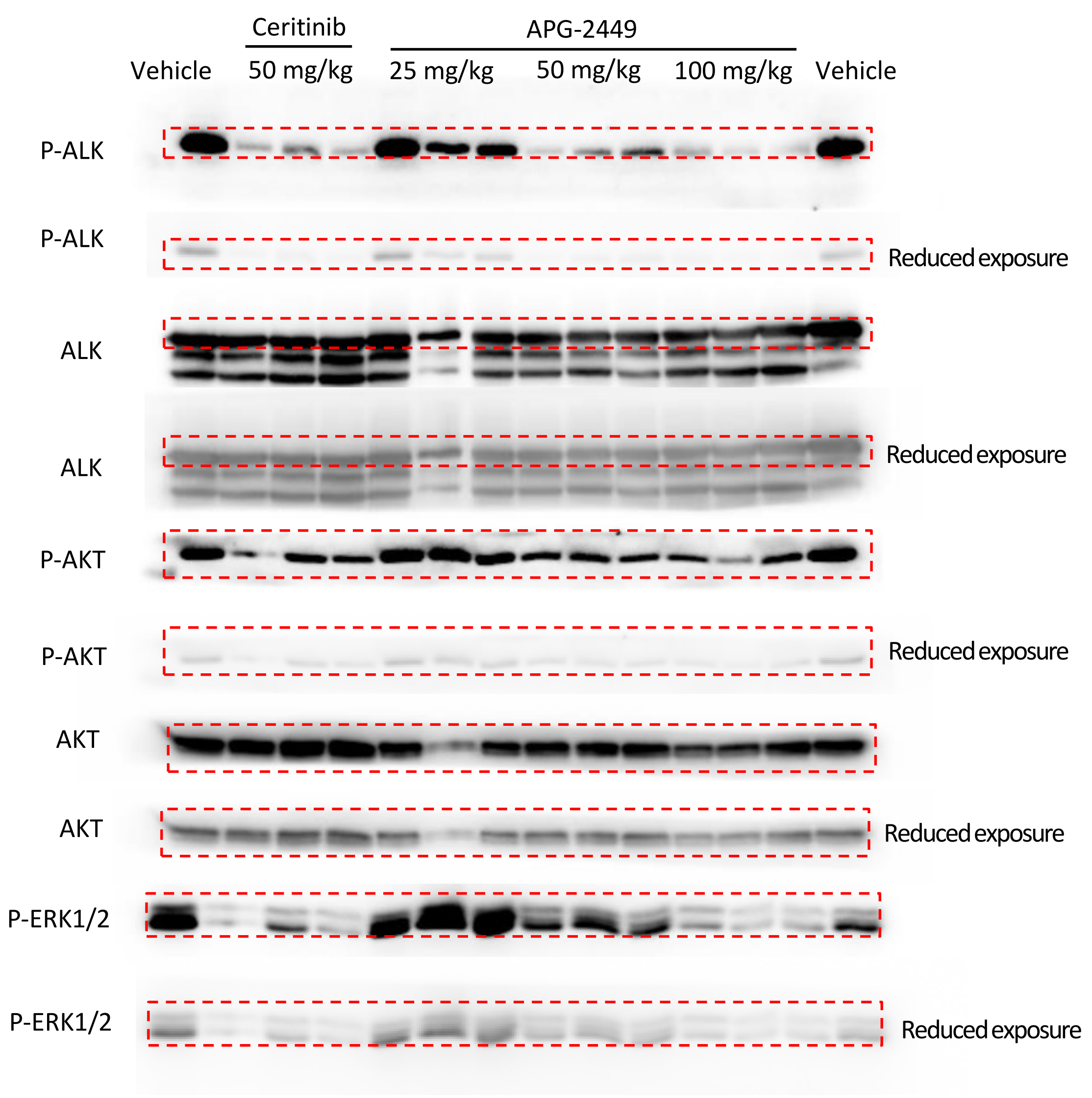

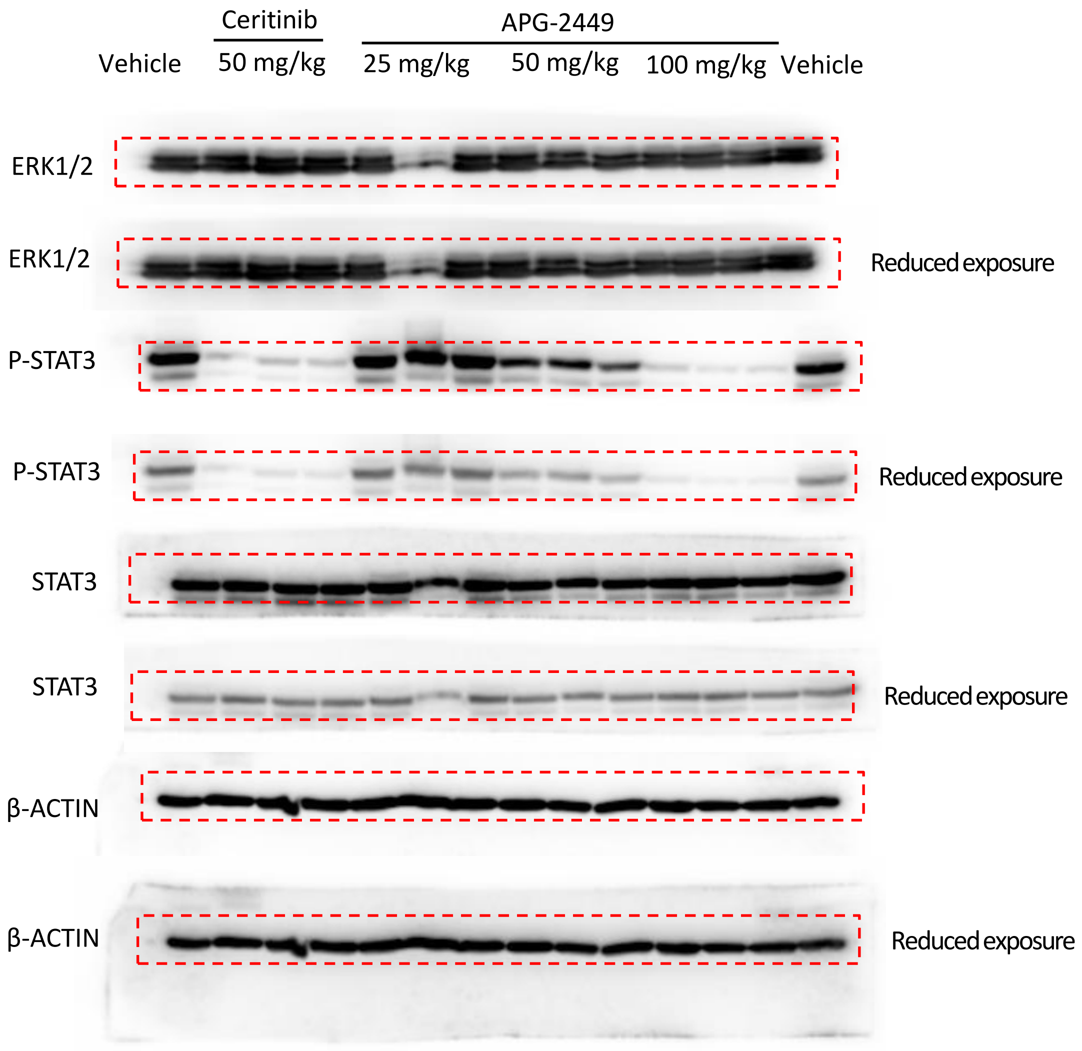

Original images for Figure 2F

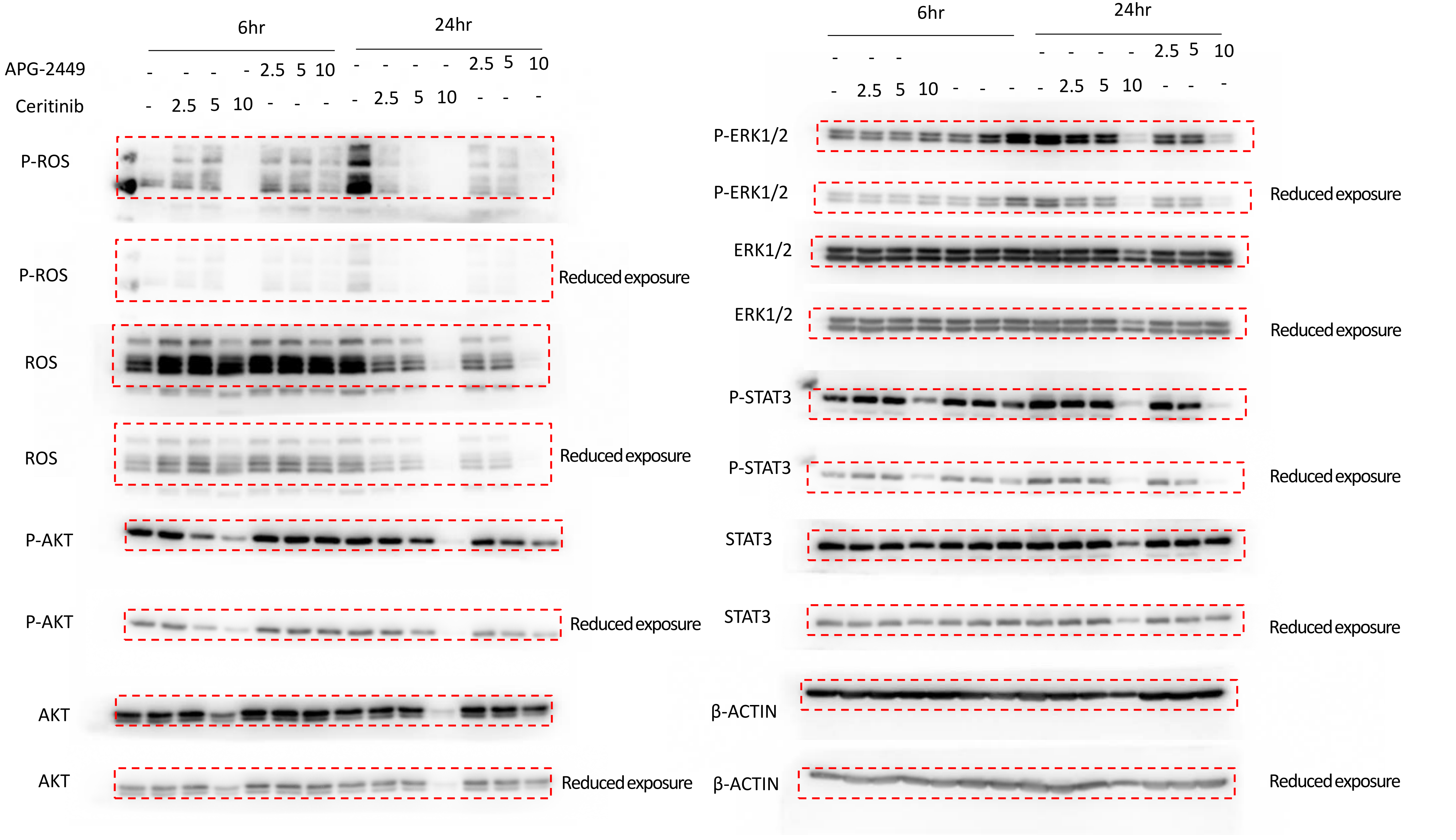

Original images for Figure 4A

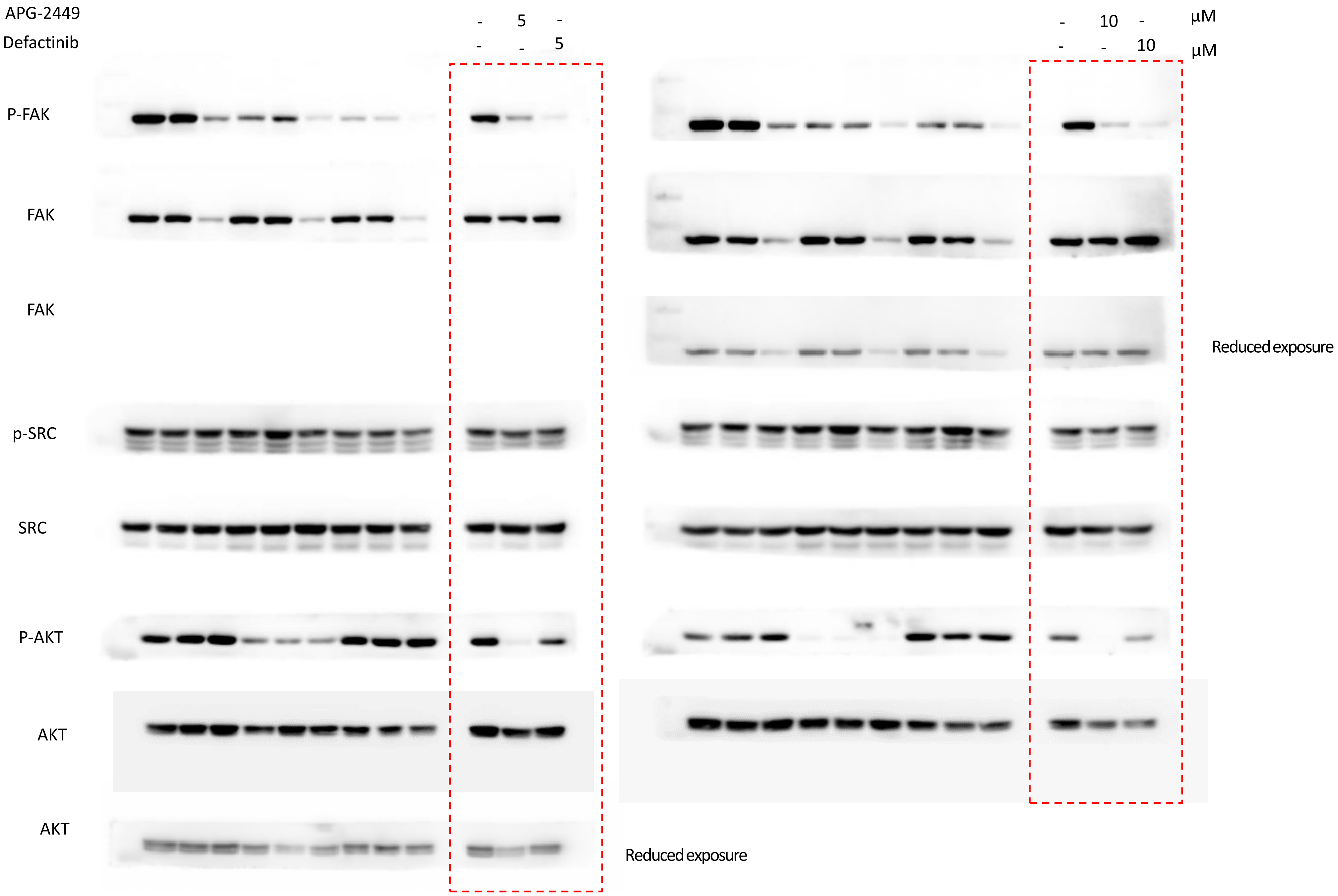

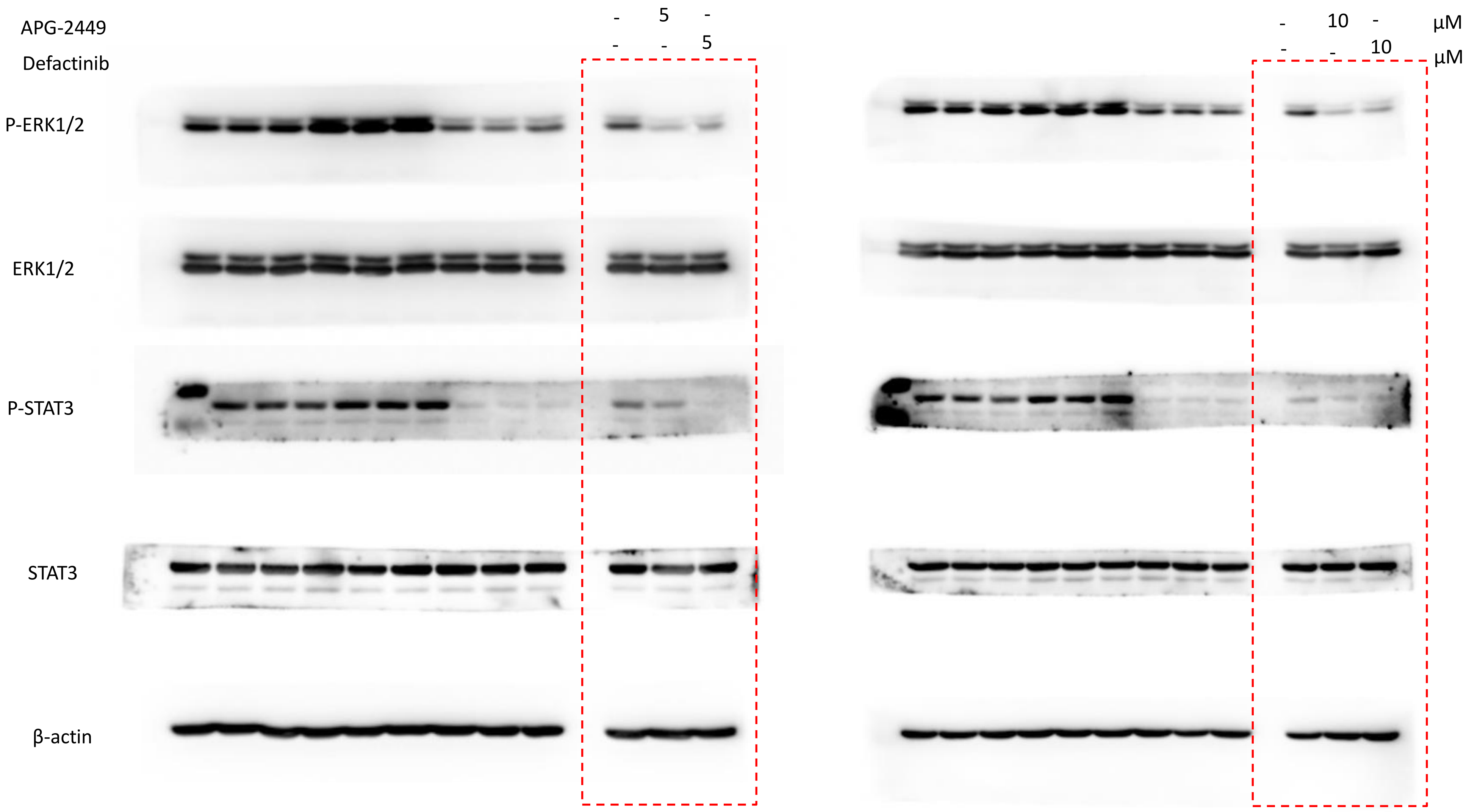
APG-2449
Defactinib
- 5 -
- - 5
- 10 - μM
- - 10 μM
P-ERK1/2
ERK1/2
P-STAT3
STAT3
β-actin

Original images for Figure 4F

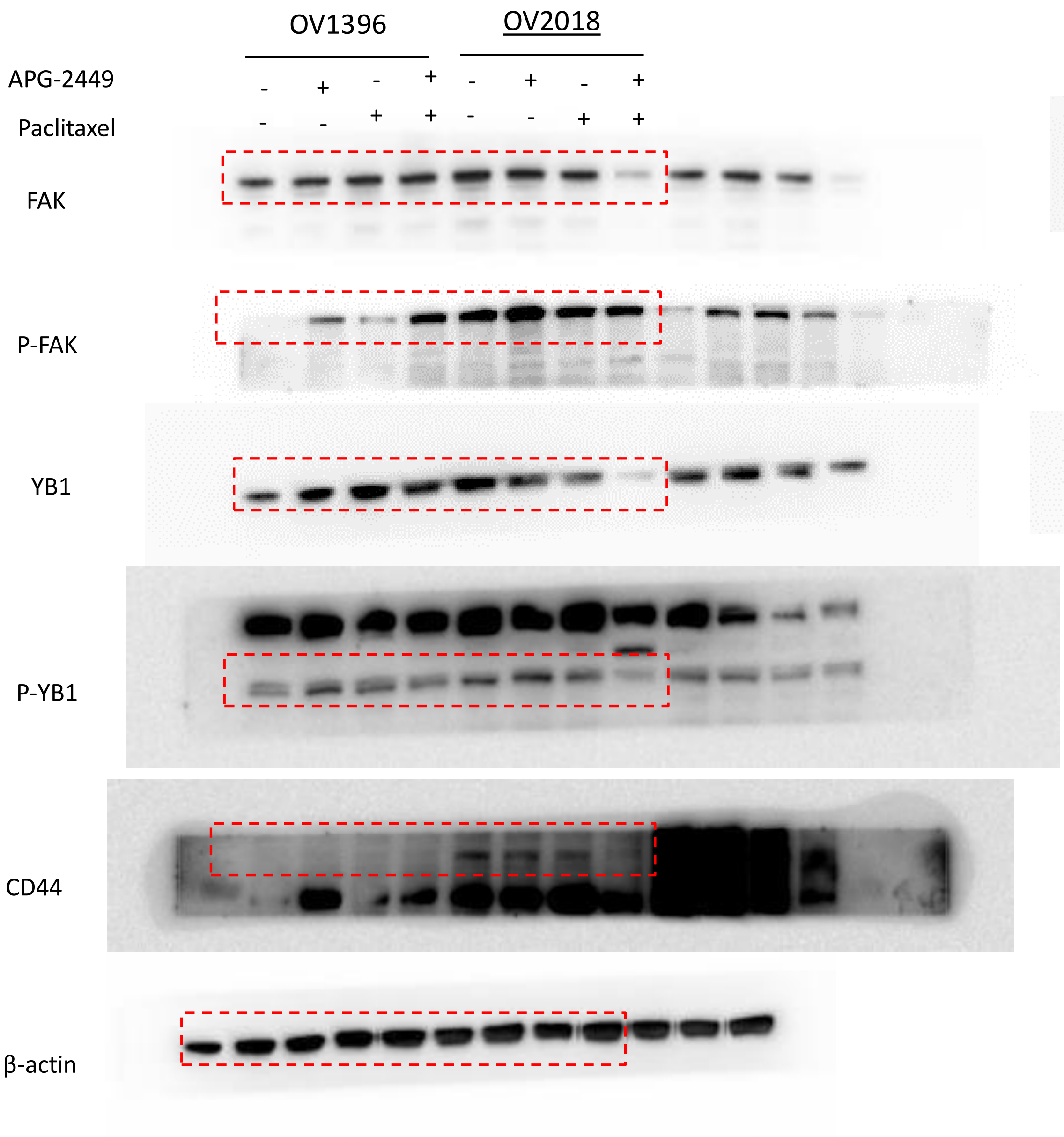

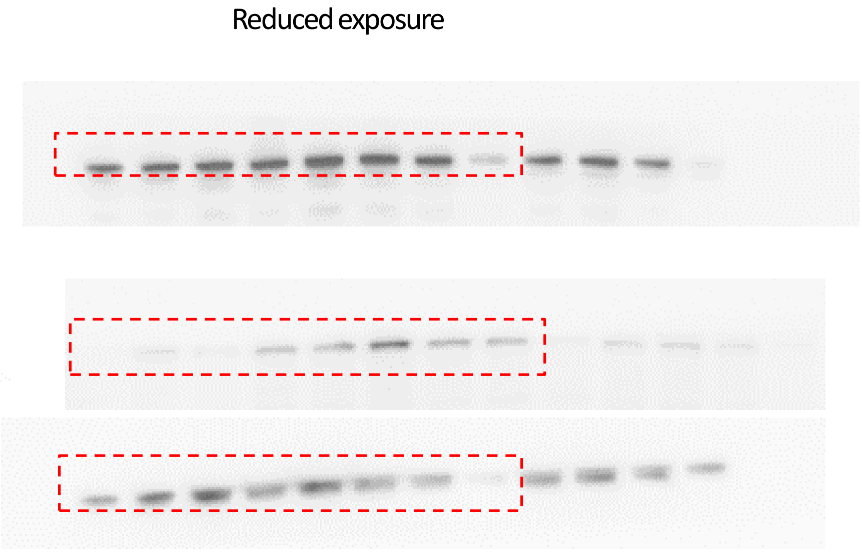

Original images for Figure 5E

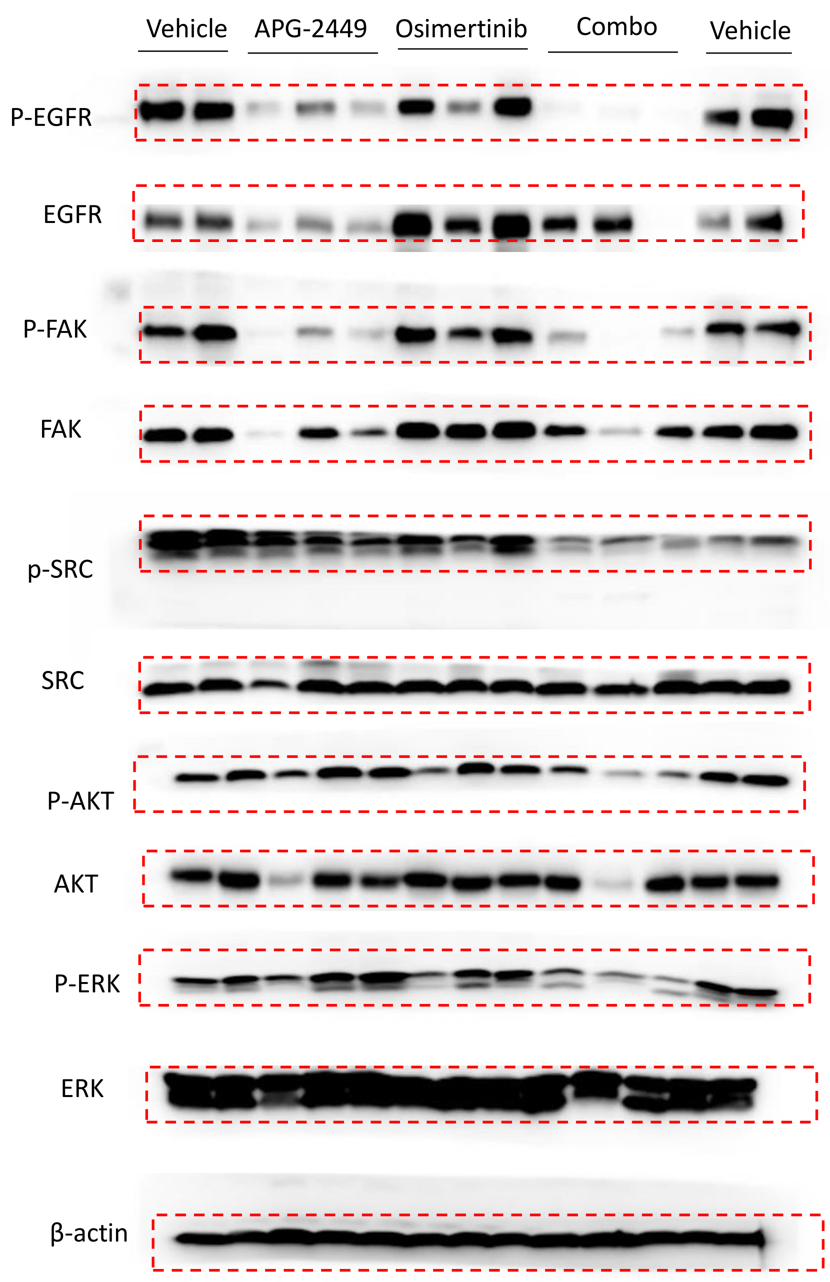

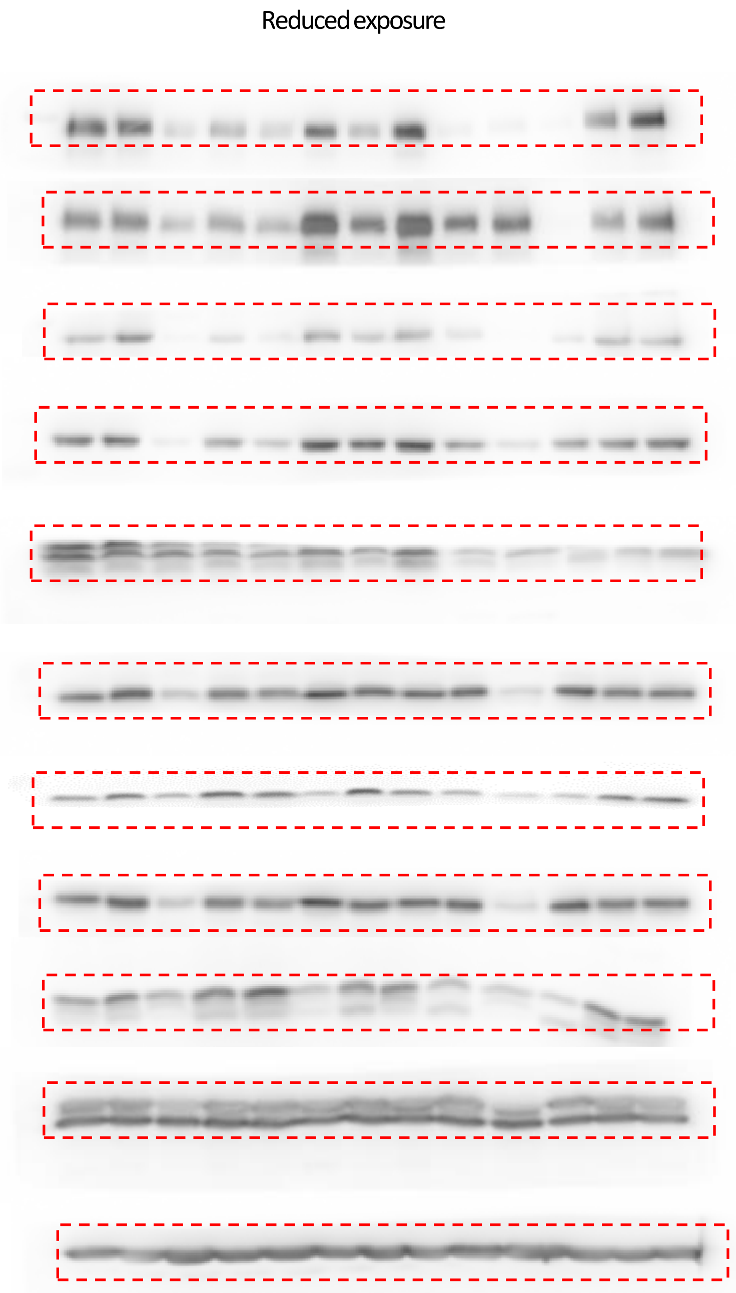

Original image for Supplementary Fig S2A

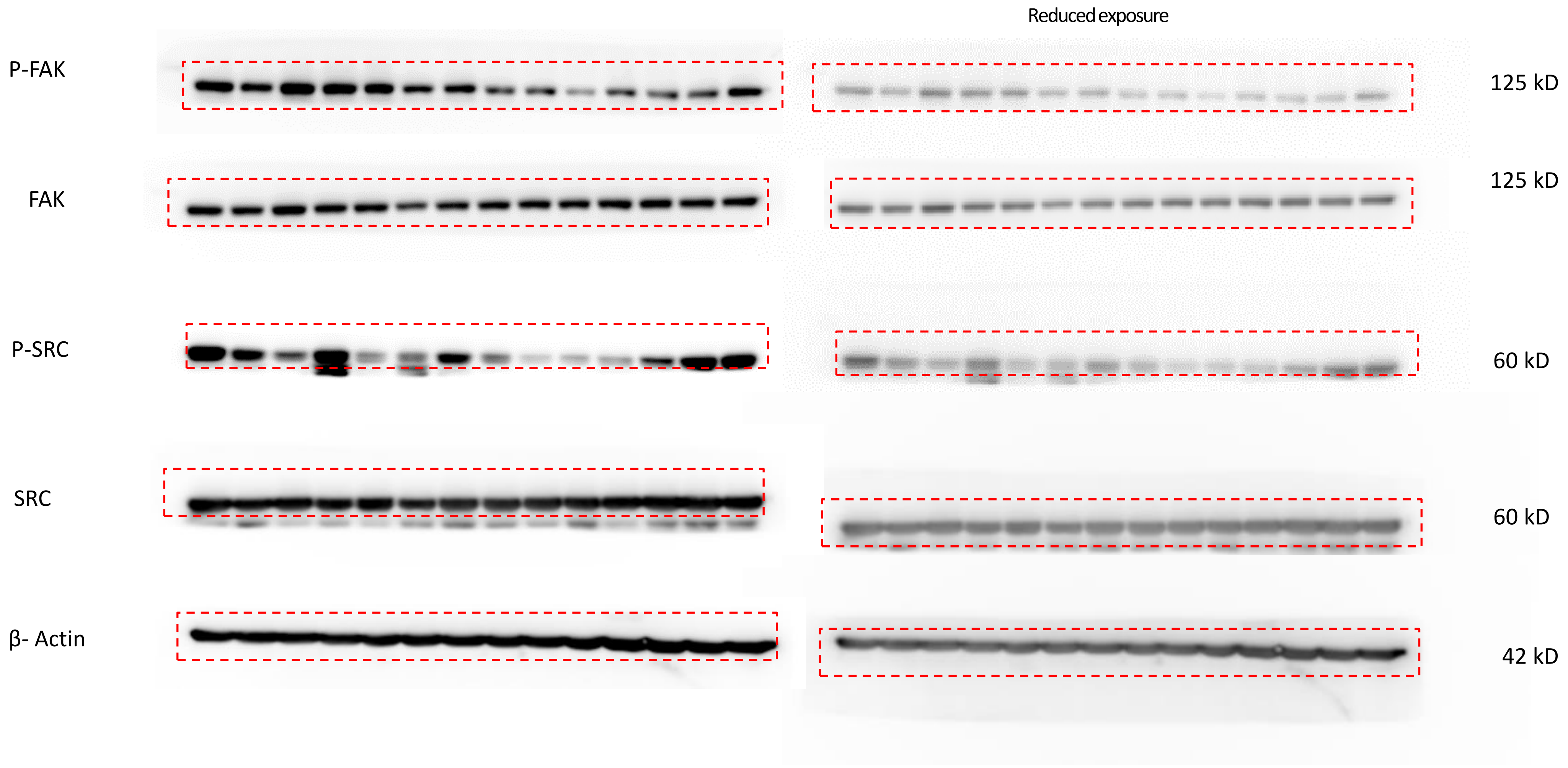

Supplement: Supplementary file 1 — Additional file 1. [file 12885_2022_9799_MOESM1_ESM.pdf]
