# Supplementary material for: Discovery of a novel ALK/ROS1/FAK inhibitor, APG-2449, in preclinical non-small cell lung cancer and ovarian cancer models
Source: BMC Cancer. 2022 Jul 11;22:752. doi: 10.1186/s12885-022-09799-4 (PMC9277925; doi:10.1186/s12885-022-09799-4)
Supplement: Supplementary file 2 — Additional file 2: Figure S1. Inhibition curves of ALK kinase activity by APG-2449 and reference compounds alectinib and ceritinib assessed by LANCE TR-FRET assay. Wild-type (wt) ALK (A), ALK L1196M mutant (B), ALK F1197M mutant (C), ALK G1269A mutant (D), ALK S1206Y mutant (E) and ALK G1202R mutant (F). LANCE TR-FRET, lanthanide chelate excite time-resolved fluorescence resonance energy transfer. Figure S2. Antitumor activity of APG-2449 in ALK-positive xenograft tumor models in mice. (A) Assessment of changes in body weight (%) of mice bearing H3122 xenograft tumors as shown in Fig. 2A. (B) Independent repeat experiment in NSCLC PDX LD1–0006-390,637 (treated for 3 weeks, n = 3–5 per treatment group) as shown in Fig. 3D. Figure S3. Combination of APG-2449 and paclitaxel inhibits tumor growth in ovarian cancer xenograft models in mice. (A) Western blotting analysis of FAK downstream signaling in PA-1 tumors collected from experiment as shown in Fig. 4B. (B) Changes in body weights (%) of OVCAR-3 xenograft-bearing mice as shown in Fig. 4C. IHC staining (C) and quantitation of staining intensity (D) of CD44, E-Cadherin (E-Cad), FAK, and p-FAK in untreated PDX tumors shown in Fig. 4D. Figure S4. Enhancement of osimertinib-mediated tumor suppression by APG-2449 in NSCLC. (A) Repeated efficacy study using a higher dose of osimertinib (15 mg/kg) in NSCLC PDX LD1–0006-215,676 (vehicle and APG-2449 groups were treated for 16 days and other groups for 23 days, n = 8–10 per treatment group). (B) Changes in body weights (%) of tumor-bearing mice in the experiment shown in A. (C) Protein expression levels shown in Fig. 5E were quantitated and shown as mean ± SEM relative to the loading control β-actin or total proteins where phosphorylated proteins were assessed (n = 3 mice per treatment group). *p < 0.05, **p < 0.01 vs. vehicle control. Supplementary Table S1. Antibodies used for western blotting. Supplementary Table S2. Genetic characteristics of ovarian cancer PDX models. [file 12885_2022_9799_MOESM2_ESM.docx]

**Supplementary Materials**


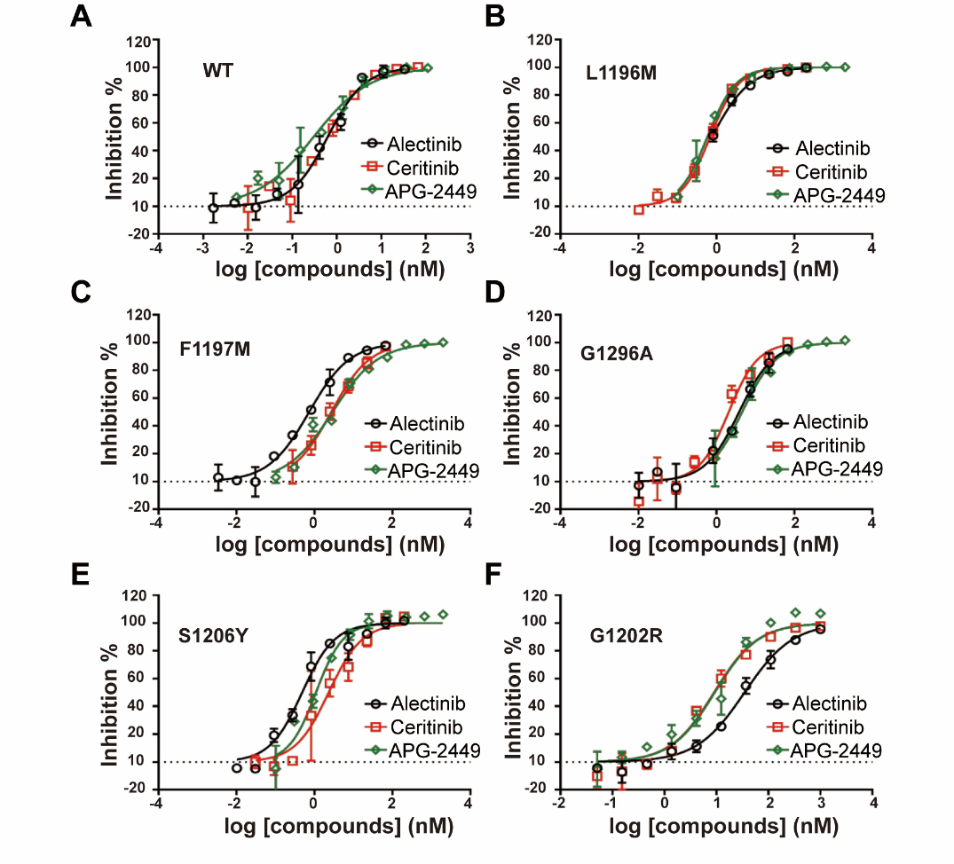


## Figure S1. Inhibition curves of ALK kinase activity by APG-2449 and reference compounds alectinib and ceritinib assessed by LANCE TR-FRET assay. Wild-type (*wt*) *ALK* (A), *ALK ^L1196M^* mutant (B), *ALK ^F1197M^* mutant (C), *ALK ^G1269A^* mutant (D), *ALK ^S1206Y^* mutant (E) and *ALK ^G1202R^* mutant (F). LANCE TR-FRET, lanthanide chelate excite time-resolved fluorescence resonance energy transfer


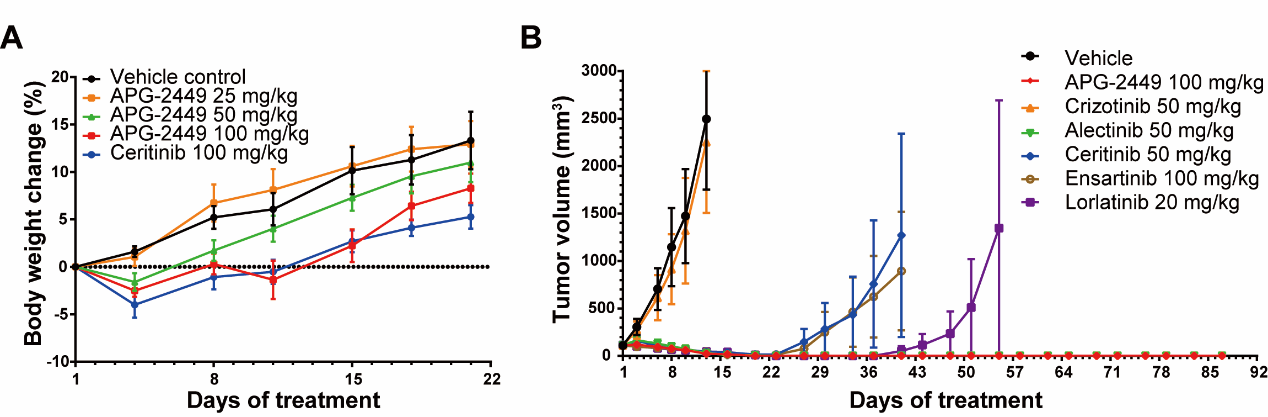


## Figure S2. Antitumor activity of APG-2449 in ALK-positive xenograft tumor models in mice.

## (A) Assessment of changes in body weight (%) of mice bearing H3122 xenograft tumors as shown in Figure 2A. (B) Independent repeat experiment in NSCLC PDX LD1-0006-390637 (treated for 3 weeks, n = 3-5 per treatment group) as shown in Figure 3D.


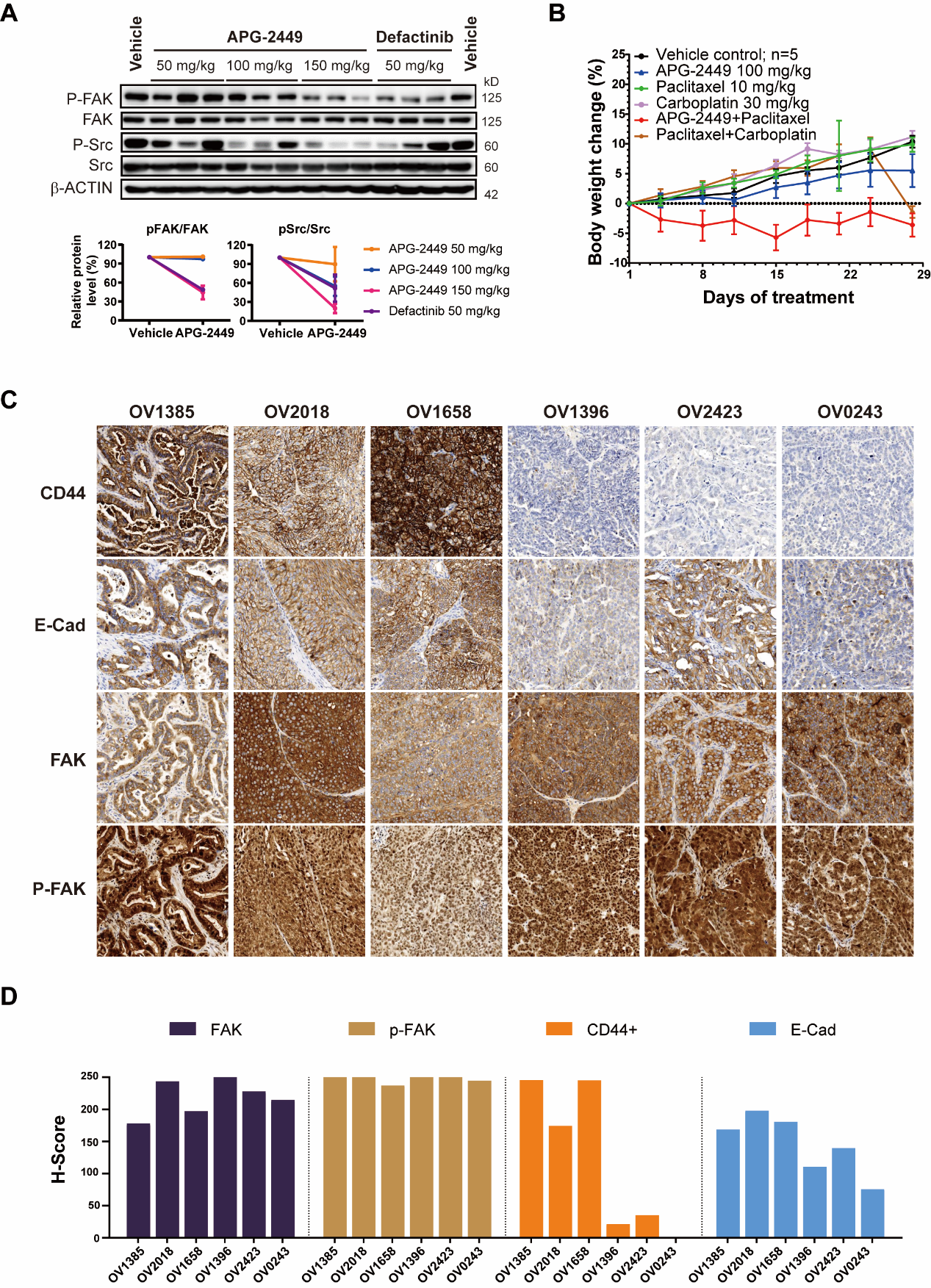


## Figure S3. Combination of APG-2449 and paclitaxel inhibits tumor growth in ovarian cancer xenograft models in mice. (A) Western blotting analysis of FAK downstream signaling in PA-1 tumors collected from experiment as shown in Figure 4B. (B) Changes in body weights (%) of OVCAR-3 xenograft-bearing mice as shown in Figure 4C. IHC staining (C) and quantitation of staining intensity (D) of CD44, E-Cadherin (E-Cad), FAK, and p-FAK in untreated PDX tumors shown in Figure 4D.


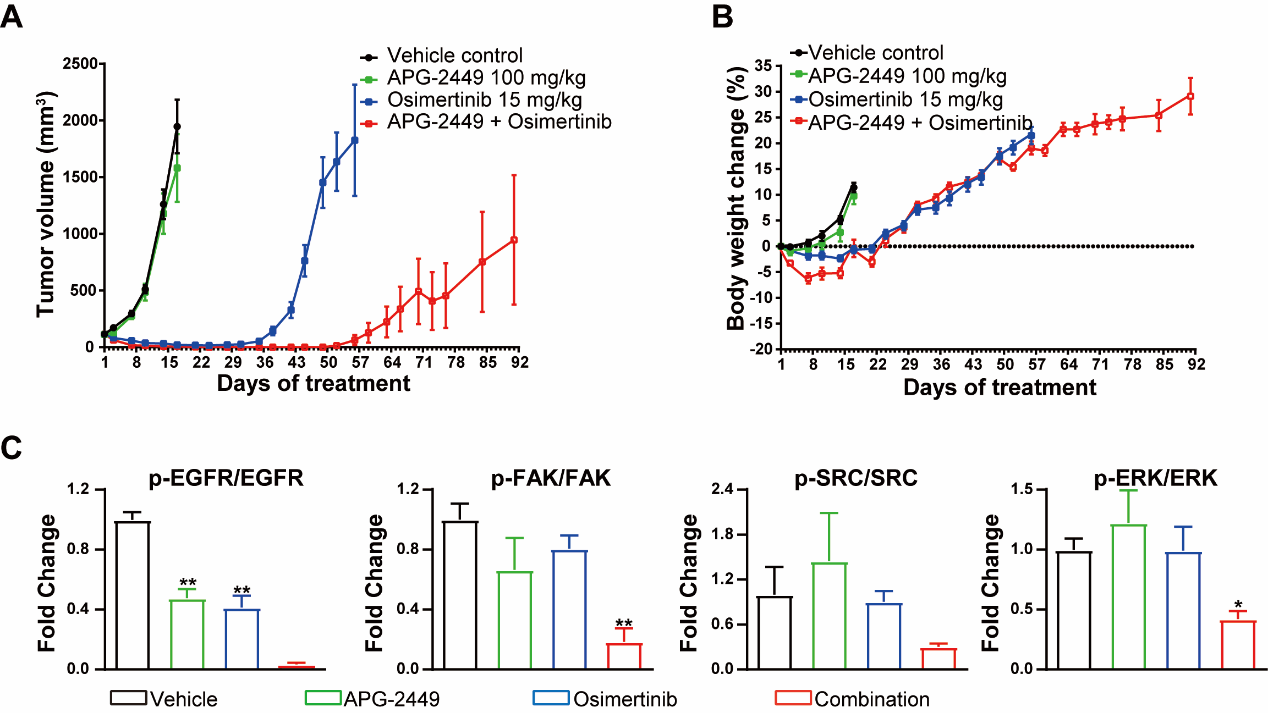


## Figure S4. Enhancement of osimertinib-mediated tumor suppression by APG-2449 in NSCLC.

## (A) Repeated efficacy study using a higher dose of osimertinib (15 mg/kg) in NSCLC PDX LD1-0006-215676 (vehicle and APG-2449 groups were treated for 16 days and other groups for 23 days, n = 8-10 per treatment group). (B) Changes in body weights (%) of tumor-bearing mice in the experiment shown in A. (C) Protein expression levels shown in Figure 5E were quantitated and shown as mean ± SEM relative to the loading control β-actin or total proteins where phosphorylated proteins were assessed (n = 3 mice per treatment group). **p* < 0.05, ***p* < 0.01 vs. vehicle control.

## Supplementary Table S1. Antibodies used for western blotting.

| Antibody | Manufacturer | Cat. # |
| --- | --- | --- |
| p-FAK (Tyr397) | CST | 8556 |
| FAK | CST | 13009 |
| p-ALK (Tyr1604) | CST | 3347s |
| ALK | CST | 31278s |
| p-SRC (Tyr416) | CST | 2109 |
| SRC | CST | 6943 |
| p-AKT (Ser473) | CST | 4060 |
| AKT | CST | 4685 |
| p-ERK1/2 (Thr202/Tyr204) | CST | 4377 |
| ERK | CST | 4695 |
| p-STAT3 (Tyr705) | CST | 9145 |
| STAT3 | CST | 9139 |
| p-ROS1 (Tyr2274) | CST | 3078 |
| ROS1 | CST | 3287 |
| p-EGFR (Y1068) | CST | 3777 |
| EGFR | CST | 4267 |
| p-YB1 (Ser102) | CST | 2900 |
| YB1 | CST | 8475 |
| CD44 | CST | 37259 |
| β-actin | CST | 4970 |
| goat anti-mouse-HRP | Yeasen | 33201ES60 |
| goat anti-rabbit-HRP | Yeasen | 33101ES60 |

CST, Cell Signaling Technology (Danvers, MA USA); HRP, horseradish peroxidase; YB, Y-box protein; Yeasen, Yeasen Biotechnology (Shanghai) Co., Ltd.

## Supplementary Table S2. Genetic characteristics of ovarian cancer PDX models.

| PDX | Treatment  (Days) | Carboplatin  (T/C %) | *BRCA1* | *BRCA2* | *TP53* | PTK2  (CNV) | PTK2  Log2 (FPKM) |
| --- | --- | --- | --- | --- | --- | --- | --- |
| OV1385 | 35 | 51.5 | WT | WT | mut | 2.88 | 6.00 |
| OV2018 | 35 | 113.2 | mut | WT | - | 5.56 | 7.19 |
| OV1658 | 19 | 68.4 | WT | WT | WT | 2.11 | 5.21 |
| OV1396 | 43 | 65.3 | WT | WT | mut | 6.81 | 7.24 |
| OV2423 | 28 | 47.8 | mut | WT | mut | 4.93 | 6.08 |
| OV0243 | 36 | 0.0 | WT | mut | mut | 5.07 | 6.31 |
| Range (n=78) | ─ | ─ | ─ | ─ | ─ | 1.36-6.81 | 2.66-7.24 |
| Mean  (n=78) | ─ | ─ | ─ | ─ | ─ | 3.17 | 5.54 |

*BRCA*, breast cancer gene; CNV, copy number variation; FPKM, fragments per kilobase million; mut, mutant; PTK, protein tyrosine kinase; *TP53*, tumor protein 53 (tumor suppressor) gene; WT, wild-type.
